# The potential effect of improved provision of rabies post-exposure prophylaxis in Gavi-eligible countries: a modelling study

**DOI:** 10.1016/S1473-3099(18)30512-7

**Published:** 2019-01

**Authors:** WHO Rabies Modelling Consortium, Katie Hampson, Francesco Ventura, Rachel Steenson, Rebecca Mancy, Caroline Trotter, Laura Cooper, Bernadette Abela-Ridder, Lea Knopf, Moniek Ringenier, Tenzin Tenzin, Sowath Ly, Arnaud Tarantola, Ronelngar Moyengar, Assandi Oussiguéré, Bassirou Bonfoh, DH Ashwath Narayana, Mysore Kalappa Sudarshan, Matthew Muturi, Athman Mwatondo, Gati Wambura, Soa Fy Andriamandimby, Laurence Baril, Glenn T Edosoa, Abdallah Traoré, Sarah Jayme, Johann Kotzé, Amila Gunesekera, Nakul Chitnis, Jan Hattendorf, Mirjam Laager, Monique Lechenne, Jakob Zinsstag, Joel Changalucha, Zac Mtema, Ahmed Lugelo, Kennedy Lushasi, Onphirul Yurachai, Charlotte Jessica E. Metcalf, Malavika Rajeev, Jesse Blanton, Galileu Barbosa Costa, Nandini Sreenivasan, Ryan Wallace, Deborah Briggs, Louise Taylor, Samuel M. Thumbi, Nguyen Thi Thanh Huong

## Abstract

**Background:**

Tens of thousands of people die from dog-mediated rabies annually. Deaths can be prevented through post-exposure prophylaxis for people who have been bitten, and the disease eliminated through dog vaccination. Current post-exposure prophylaxis use saves many lives, but availability remains poor in many rabies-endemic countries due to high costs, poor access, and supply.

**Methods:**

We developed epidemiological and economic models to investigate the effect of an investment in post-exposure prophylaxis by Gavi, the Vaccine Alliance. We modelled post-exposure prophylaxis use according to the status quo, with improved access using WHO-recommended intradermal vaccination, with and without rabies immunoglobulin, and with and without dog vaccination. We took the health provider perspective, including only direct costs.

**Findings:**

We predict more than 1 million deaths will occur in the 67 rabies-endemic countries considered from 2020 to 2035, under the status quo. Current post-exposure prophylaxis use prevents approximately 56 000 deaths annually. Expanded access to, and free provision of, post-exposure prophylaxis would prevent an additional 489 000 deaths between 2020 and 2035. Under this switch to efficient intradermal post-exposure prophylaxis regimens, total projected vaccine needs remain similar (about 73 million vials) yet 17·4 million more people are vaccinated, making this an extremely cost-effective method, with costs of US$635 per death averted and $33 per disability-adjusted life-years averted. Scaling up dog vaccination programmes could eliminate dog-mediated rabies over this time period; improved post-exposure prophylaxis access remains cost-effective under this scenario, especially in combination with patient risk assessments to reduce unnecessary post-exposure prophylaxis use.

**Interpretation:**

Investing in post-exposure vaccines would be an extremely cost-effective intervention that could substantially reduce disease burden and catalyse dog vaccination efforts to eliminate dog-mediated rabies.

**Funding:**

World Health Organization.

## Introduction

Timely post-exposure prophylaxis prevents the fatal onset of rabies, which causes an estimated 60  000 human deaths each year, mostly in Africa and Asia and among children.^1^, ^2^ Domestic dogs are responsible for 99% of human cases.^3^ Although mass dog vaccination is required for elimination of dog-mediated rabies, the disease burden could be substantially reduced through improved access to post-exposure prophylaxis.

WHO recommendations for rabies post-exposure prophylaxis have been updated in line with new evidence.^4^ Procedures depend on the type of contact with the suspect rabid animal, with administration of rabies immunoglobulin recommended for high-risk exposures. Intradermal multisite vaccination regimens have been developed, which are more economical than intramuscular administration because they use reduced vaccine volumes.^4^ While the use of post-exposure prophylaxis at current levels saves many lives,^2^ access to post-exposure prophylaxis is poor in many parts of the world, particularly rural areas where most rabies exposures occur. Even if people who have been bitten get to a treatment centre and post-exposure prophylaxis is available, its cost is often unaffordable.

A global framework to reach zero human deaths from dog-mediated rabies by 2030 was developed by WHO and partners in 2015.^3^ A strategic plan covering human and animal vaccine demand was developed for the implementation of this framework.^5^ In 2016, WHO established a Strategic Advisory Group of Experts Working Group on rabies vaccines and rabies immunoglobulins,^6^ aiming to increase the public health effect of rabies biologics through practical and feasible recommendations. This effort resulted in a recommendation of a dose-sparing abridged 1-week intradermal regimen, requiring only three clinic visits, and guidance for more prudent use of rabies immunoglobulin.^4^

**Research in context**

**Evidence before this study**

We searched PubMed for papers published from Jan 1, 1980, to May 31, 2018, with the terms “rabies” AND “burden” AND (“global” OR “Africa” OR “Asia”) and identified two previous modelling studies that suggested around 60 000 deaths from rabies occur each year. Post-exposure prophylaxis after a suspected rabies exposure is a safe and effective way of preventing human deaths from rabies and has long been promoted by WHO. However, human deaths from rabies remain high in many low-income and middle-income countries because access to post-exposure prophylaxis is poor. Although mass dog vaccination is required for elimination of dog-mediated rabies, improved provision and use of post-exposure prophylaxis would also prevent deaths. Dose-sparing intradermal vaccine regimens are known to be much more economical than intramuscular regimens but have not been adopted everywhere.

**Added value of this study**

This is the first paper to consider the effect of an investment in rabies post-exposure prophylaxis across all 67 countries that are, or have previously been, Gavi-eligible and where rabies is endemic. Although data are still scarce in many areas, we used information from studies supported by the Gavi learning agenda. Improving provision of rabies vaccines for post-exposure prophylaxis is a highly cost-effective intervention that could prevent an additional 489 000 deaths from rabies from 2020 to 2035. Our analyses suggest that investments should prioritise vaccines for post-exposure prophylaxis rather than rabies immunoglobulin, which is costly and has more marginal health benefits. Even with expanding dog vaccination efforts and associated reductions in the risk of human exposure over the 2020–35 time period, improved access to post-exposure prophylaxis remains a highly cost-effective intervention.

**Implications of all the available evidence**

Increasing timely access to rabies vaccines for post-exposure prophylaxis, free at point-of-care, would save many lives, is highly cost-effective, and is feasible under the current vaccine production capacity, with the switch to the dose-sparing abridged 1-week intradermal regimen. In combination with scaled-up mass dog vaccination, an investment to improve access to post-exposure prophylaxis could be transformative for rabies prevention and could catalyse the global campaign Zero by 30 to eliminate human deaths from dog-mediated rabies.

Enhancing access to rabies post-exposure prophylaxis was considered by Gavi, the Vaccine Alliance, in their vaccine investment strategy in 2008 and 2013.^7^ In 2013, post-exposure prophylaxis was estimated to avert almost 200 000 future deaths between 2015 and 2030 at a low cost per death averted, and that investment could stimulate the shift to more economical intradermal vaccination.^7^ However, knowledge gaps were recognised and observational studies were recommended to reduce uncertainties about implementation feasibility. Subsequently, Gavi supported field studies on rabies burden, treatment seeking, post-exposure prophylaxis compliance, and vaccine efficacy as part of their learning agenda. Gavi will reconsider the rabies vaccine investment case in December, 2018.

We model the epidemiological and economic effect of changes in policy and practice for the provision of post-exposure prophylaxis, which Gavi investment could support.

## Methods

Given inadequate rabies surveillance, studies of human rabies deaths rely upon model-derived estimates.^8^ We use an economic model of post-exposure prophylaxis demand linked to an epidemiological model characterising rabies dynamics in domestic dog populations (the primary reservoir).

We included countries that are currently (n=46), or have ever been, Gavi-eligible (n=67) and are endemic for rabies (appendix), henceforth denoted as Gavi-46 and Gavi-67. We did all analyses using R version 3.4.1. We used GATHER^9^ and CHEERS^10^ checklists to improve reporting quality (appendix).

### Economic model

**Figure 1:**
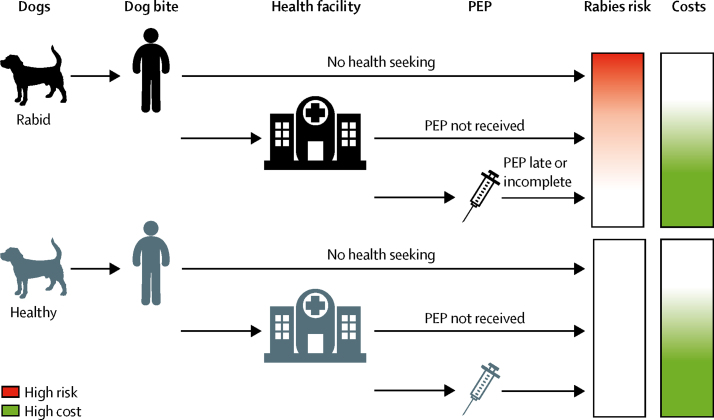
Decision tree covering rabies exposure, health seeking, and health outcomes, including death due to rabies or prevention through PEP PEP provided to individuals bitten by healthy animals results in costs but does not avert deaths. For the full decision tree, see appendix. PEP=post-exposure prophylaxis.

We adopted the static decision tree (characterised in figure 1; appendix), informed by literature-derived parameter estimates and available data.

We took the perspective of the health provider and considered direct medical costs only. Currently, individuals bear post-exposure prophylaxis costs in many countries (appendix); with investment, post-exposure prophylaxis costs would be borne by Gavi with cofinancing from national governments. Health benefits were measured in terms of deaths and disability-adjusted life-years (DALYs) averted; rabies is universally fatal and we did not consider acute disability so DALYs are equal to years of life lost. Individuals in the model were aged 0–99 years. The time horizon was 2020–35 and our analysis accounts for human population growth over this timescale. We converted all costs to US$, 2016, using inflation indicators (Consumer Price Index) from the World Bank. All scenarios were run with discounting at 3% (0% as alternative). We calculated the cost per death and per DALYs averted and, as appropriate, incremental costs and benefits.

### Epidemiological model

Human rabies exposures depend on dog rabies incidence and rabid dog biting behaviour. We used a stochastic dynamic transmission model to characterise rabies in dog populations accounting for the effect of mass dog vaccination. Coverage achieved wanes between vaccination campaigns because of turnover in the dog population, so we incorporate dog demography. We ignore wildlife, which have a negligible role in maintenance of canine rabies.^11^ We model an endemic scenario with and without mass dog vaccination to generate typical dog rabies incidence and trajectories to elimination (appendix).

### Scenarios

We investigated the following scenarios (assumptions detailed in the panel): (1) status quo, with countries delivering post-exposure prophylaxis according to current provision rates and practices; (2) increased post-exposure prophylaxis access, with free vaccination following the dose-sparing abridged 1-week intradermal regimen^4^ and improved health seeking, vaccine provision, and post-exposure prophylaxis completion, considering a base case (2a), low variant (2b), and high variant (2c); (3) as 2, with provision of rabies immunoglobulin, as per the latest recommendations;^4^ (4) declining incidence due to scaling up mass dog vaccination (currently negligible in most Gavi-eligible countries), with post-exposure prophylaxis provided according to the status quo (4a), with improved post-exposure prophylaxis access (4b with base case parameter values), and as with the previous point, with integrated bite case management (IBCM) to reduce unnecessary post-exposure prophylaxis for people bitten by healthy animals (4c).^12^, ^13^

Panel **Parameters and assumptions for modelling scenarios**

We used biologically-defined constants for the probability of developing rabies following exposure, p_infect_, and of post-exposure prophylaxis (PEP) preventing rabies, p_prevent_ (appendix). Under the status quo (scenario 1) we used country-specific data from health facility use and Gavi Learning Agenda studies to inform parameters for health seeking and PEP provision (appendix gives country-specific estimates and gives cluster averages used when no country-specific data were available). In scenarios with improved PEP access (scenarios 2 and 3), we modelled increased health seeking, PEP provision, and compliance, as detailed here, according to timelines from the Global Strategic Plan (appendix). Dog vaccination is modelled in scenario 4, with Integrated Bite Case Management (IBCM) to reduce unnecessary PEP use for persons bitten by healthy dogs included in scenario 4c.

**p**_seek|rabid_

Probability: seeking care when bitten by suspect rabid dog.

Change with improved PEP access: initial increase of 0·1 from status quo in scenario 2a, base case (0·05 increase in scenario 2b low variant, 0·15 in scenario 2c high variant), with 0·03 increase per year thereafter to a cap of 0·9 in the base case (0·85 in scenario 2b, 0·95 in scenario 2c)

**p**_seek|healthy_

Probability: seeking care when bitten by healthy dog.

Change with improved PEP access: initial increase of 0·1 from status quo in scenario 2a, base case (0·05 increase in scenario 2b, 0·15 in scenario 2c), to a cap of 1 in all scenarios; no incremental increases after introduction.

**p**_receive_

Probability: receiving PEP if treatment sought.

Change with improved PEP access: initial increase of 0·1 from status quo in scenario 2a, base case (0·05 examined as a scenario 2b and 0·15 as a scenario 2c), with 0·03 increase per year thereafter to a cap of 0·93 base case (0·88 scenario 2b, 0·98 scenario 2c). Under scenario 4c with IBCM, p_receive_ was reduced to 0·5 for healthy dog bites on implementation of the intervention and was reduced further to 0·1 after elimination (zero dog rabies cases).

**p**_complete_

Probability: completing regimen.

Change with improved PEP access: **i**nitial increase of 0·1 in scenario 2a, base case (0·05 in scenario 2b, 0·15 in scenario 2c), with 0·03 increase per year thereafter to a cap of 0·8 base case (0·75 scenario 2b, 0·85 scenario 2c).

We simulated staggered implementation as described in the Global Strategic Plan Zero by 30 (appendix), assuming improved post-exposure prophylaxis access from 2020 onwards for phase 1 countries (n=21), from 2022 for phase 2 countries (n=33), and from 2026 for phase 3 countries (n=13). On implementation, we adjusted health seeking and post-exposure prophylaxis provision parameters and modelled incremental changes thereafter (panel).

### Data sources

We developed a data template and sought information from multiple sources (appendix). Data from contact tracing and health-care use studies were obtained from partners in Tanzania, Madagascar, Sri Lanka, and Kenya. Partners in Chad, Côte d'Ivoire, Mali, Uganda, Haiti, Cameroon, Cambodia, Bhutan, Vietnam, Thailand, and the Philippines completed data templates. A systematic literature review of papers on the burden of rabies published from Jan 1, 2013, to Feb 28, 2017, was completed,^2^ updating a previous review. Further country-specific searches (“country” AND “rabies”) in PubMed were completed in July 26, 2017. Subsequent relevant pub-lications were also reviewed where identified. Surveys of post-exposure prophylaxis provision were done by WHO and the US Centers for Disease Control and Prevention for 23 countries (15 of which were Gavi-eligible countries in 2018), covering vaccine administration route, regimen, cost, access, procurement, distribution, and monitoring. Follow-up by WHO ascertained whether patients in other countries pay for vaccines or are provided post-exposure prophylaxis free-of-charge from government clinics (appendix). We assumed that countries without information charged patients. Country-specific dog populations were estimated from human-to-dog ratios.^1^ Human population estimates and life expectancies for each country were taken from UN World Population Prospects 2017, medium variant.^14^ The proportions of populations in urban and rural environments were taken from the World Bank development indicators.^15^ Published age distributions of rabies cases were used.^1^ We calculated a less conservative set of DALYs based on WHO frontier life expectancy in 2030.^16^ Health-care costs (ie, health worker time for post-exposure prophylaxis delivery) by country were obtained from WHO-CHOICE estimates.^17^

### Model parameters

We assigned countries to a geographical cluster and we calculated cluster parameter values as the mean of country values in the cluster (appendix). Where country-specific parameters were not available or data were judged to be poor quality or inappropriate (eg, small numbers, biased sampling), we used cluster values.

Several parameters were considered biologically determined and were not expected to vary (appendix). We used reported estimates of the probability of infection following exposure (p_infect_), of complete and timely post-exposure prophylaxis (p_prevent1_), or incomplete or late post-exposure prophylaxis (p_prevent2_) preventing infection.^18^ For Ethiopia, where less effective nerve tissue vaccines are used, we used published data to estimate the probability that complete (p_prevent3_) or incomplete nerve tissue vaccine use (p_prevent4_) prevents infection.^19^

To capture rabies exposures, we multiplied dog rabies incidence by a per-capita transmission probability and national dog population estimates (appendix). This calculation assumes only a fraction of rabid dogs bite people (on average 0·38 people are bitten per rabid dog;^20^ appendix). Using incidence data from multiple countries of patients who have been bitten, we estimated the proportion of rabid versus healthy animals and baseline health-seeking probabilities following rabid and healthy dog bites (p_seek|rabid_, p_seek|healthy_; appendix).

### Post-exposure prophylaxis delivery

We estimated post-exposure prophylaxis use for each scenario. Vials of rabies immunoglobulin and vaccines for intradermal use contain multiple doses but opened vials must be discarded at the end of the day. A modelling analysis informed our assumptions for projected vial sharing under different clinic throughputs.^21^ We assumed use of 1 mL vials with an average of 2·2 vials per complete 1-week intradermal post-exposure prophylaxis in rural settings, 0·67 vials per complete post-exposure prophylaxis in urban settings, and 1·47 vials per incomplete post-exposure prophylaxis in rural settings and 0·45 vials in urban settings (ie, <three visits; appendix).

Immunoglobulin use in virtually all Gavi-eligible countries is negligible (with some notable exceptions—eg, Sri Lanka, India, and Bhutan); therefore, it was not included in the status quo. In scenario 3, we assume administration of rabies immunoglobulin only in urban clinics, and then only to high-risk patients—ie, with multiple or deep bites, bites to the head or hands, bites by confirmed rabid dogs, or to immunocompromised patients. Around 15% of patients fit these criteria from reviews of clinic data from different countries. We modelled 0·32 rabies immunoglobulin vials per patient on average (appendix).^21^

We used reported costs of post-exposure prophylaxis (appendix) to model the status quo and assumed that upon Gavi investment, rabies vaccine would be purchased for $5 per vial and rabies immunoglobulin at $45 per vial (similar to current prices of equine-derived immunoglobulin from the Pan American Health Organization revolving fund). We assumed fixed introductory costs of $100 000 per country to facilitate training and implementation of improved post-exposure prophylaxis access.

### Sensitivity analysis

We examined the effect of different parameters with one-way sensitivity analyses. For the principal model outputs, we ran a probabilistic sensitivity analysis taking 1000 draws from the parameter distributions (based on 95% CIs) to generate a 95% prediction interval (PI) and mean central estimate. We examined the sensitivity of results to uncertainty in parameters in the epidemiological model separately (appendix). Because the future price of post-exposure prophylaxis is unknown, we did not include it in the probabilistic sensitivity analysis, but we investigated vaccine costs of $10 and $2·5 per vial and rabies immunoglobulin costs of $20 per vial. We increased the introductory costs to $500 000 given the uncertain costs of scaling up post-exposure prophylaxis, since studies on vaccine introduction indicate that such grants might underestimate true costs.^22^

### Role of the funding source

The sponsor of the study (WHO) supported a meeting of the Rabies Modelling Consortium and WHO employees (BA-R and LK) contributed to study design, data collection, interpretation, and writing. The corresponding authors had full access to all the data in the study and final responsibility for the decision to submit for publication.

## Results

**Figure 2:**
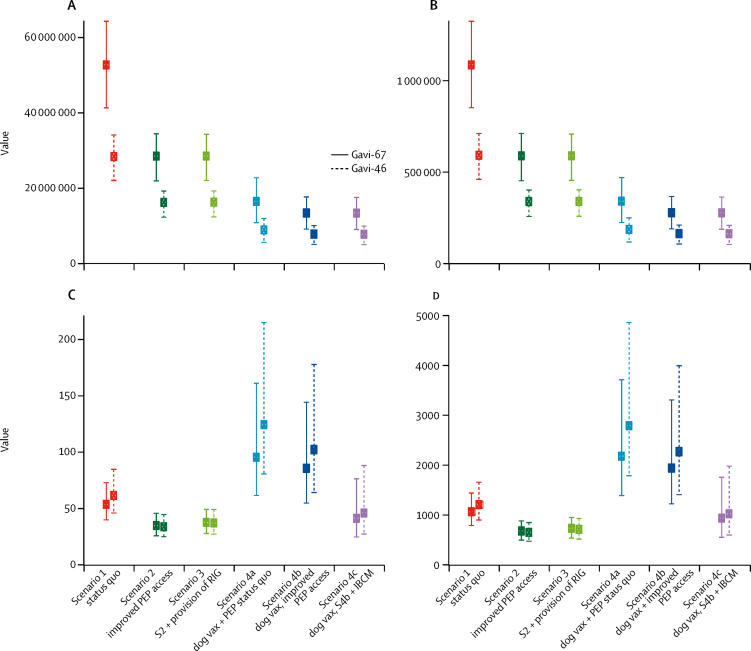
DALYs (undiscounted; A), deaths (undiscounted; B), cost per DALYs averted (discounted; C), and cost per death averted (discounted; D) from 2020 to 2035 under the modelled scenarios Estimated outcomes presented with 95% PIs. Estimates do not include costs of dog vaccinations and only show the cost-effectiveness of PEP incremental to dog vaccination. Costs of IBCM are assumed to be covered by introductory grants (together with improved PEP access). Modelling assumptions are shown in panel and results in appendix. Only the base case is shown for scenario 2—ie, scenario 2a base case. DALYs=disability-adjusted life-years. IBCM=integrated bite case management. PEP=post-exposure prophylaxis. PI=prediction interval. RIG=rabies immunoglobulin. S=scenario. Vax=vaccination.

From 2020 to 2035, we estimate that under the status quo 1 074 000 dog-mediated rabies deaths (95% PI 852 000–1 325 00) in humans will occur in the 67 endemic countries considered (figure 2; 580 000 deaths in Gavi-46 countries)—around 67 000 deaths per year. Approximately 106 000 (10%) of 1 074 000 deaths (84 200–131 000) are children younger than 5 years. Most deaths are in sub-Saharan Africa (347 000 in the east Africa cluster and 231 000 in the west Africa cluster) and Asia (464 000), with much fewer in the Americas (33 000). Country-specific burden estimates are provided in the appendix. After standardising for population growth, our estimates of deaths are similar to previous estimates^2^ in most countries (appendix). Current levels of post-exposure prophylaxis prevent approximately 56 000 deaths or 2 764 000 DALYs per year.

**Figure 3:**
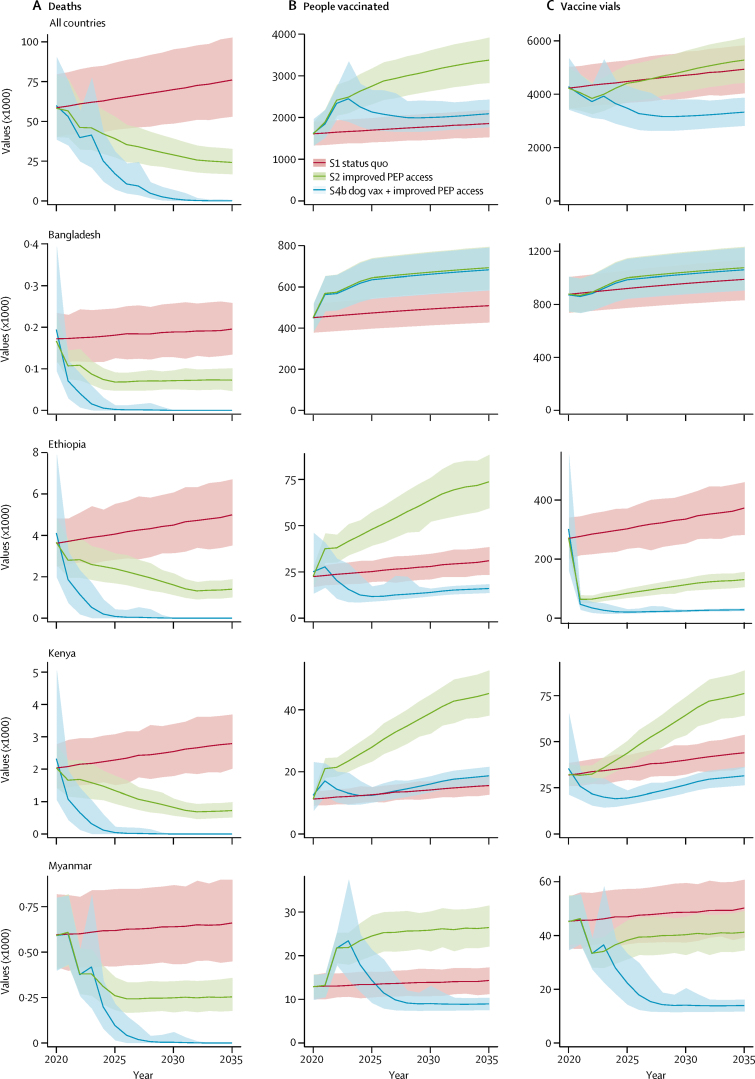
Projected outcomes under different scenarios over the 2020–35 time period Estimated outcomes per year presented with 95% PIs. (A) Human deaths from rabies (× 1000); (B) people initiating courses of PEP (× 1000); (C) vials of vaccine (× 1000) required for all 67 countries (top) and for selected countries (Bangladesh, Ethiopia, Kenya, and Myanmar) according to the status quo (scenario 1), under improved PEP access (scenario 2, base case), and with improved access to PEP vaccines concomitant with mass dog vaccination (scenario 4b). The step changes correspond to the timing of improvements in access to PEP and introduction of dog vaccination programmes. Bangladesh, Ethiopia, and Kenya are all phase 1 countries (implementation in 2020) in the Global Strategic Plan (appendix), whereas Myanmar is a phase 2 country (implementation in 2022). PEP=post-exposure prophylaxis. PI=prediction interval. S=scenario. Vax=vaccination.

With improved post-exposure prophylaxis access (scenario 2; base case) projected annual deaths and DALYs more than halved from 2020 to 2035 (table), equating to a total of 1 388 000 deaths averted or 68 010 000 DALYs averted over this period. Bite victims initiating post-exposure prophylaxis from 2020 to 2035 increase from 27·8 million under the status quo to 45·2 million (figure 3) with improved post-exposure prophylaxis access. Switching to the dose-sparing abridged 1-week intradermal regimen means that overall vaccine demand does not change very much (stays around 73 million vials at a [undiscounted] cost of about $11·1 billion). Annual costs with improved access (scenario 2; base case) would be $69·2 million in Gavi-67 or $29·3 million in Gavi-46 countries (appendix). Under the status quo, costs of post-exposure prophylaxis are mainly borne by patients (48 of 67 countries). From 2020 to 2035, assuming Gavi investment displaces personal but not government expenditure (appendix), $975·1 million (undiscounted costs) would be required to deliver improved access in all Gavi-67 countries and $403·7 million to Gavi-46 countries.

**Table:** Model results across all Gavi-67 countries projected over 2020–35 for the different scenarios

	**Scenario 1: status quo (95% PI)**	**Scenario 2a, base case: improved PEP access (95% PI)**	**Scenario 2b: low variant (95% PI)**	**Scenario 2c: high variant (95% PI)**
Rabies deaths	1·07 (0·852–1·32)	0·576 (0·453–0·711)	0·720 (0·561–0·885)	0·425 (0·333–0·522)
Rabies deaths averted	0·898 (0·704–1·11)	1·39 (1·09–1·72)	1·25 (0·971–1·54)	1·55 (1·22–1·90)
DALYs	52·1 (41·4–64·3)	27·9 (21·9–34·5)	34·9 (27·2–43·0)	20·5 (16·1–25·2)
DALYs averted	44·2 (34·7–54·6)	68·0 (53·3–84·3)	61·1 (47·6–75·5)	75·7 (59·7–93·0)
Vaccine vials used	73·5 (65·7–81·4)	73·8 (66·1–81·6)	62·2 (55·9–68·8)	86·8 (77·7–96·2)
PEP courses initiated	27·8 (24·6–31·0)	45·2 (40·5–50·0)	38·2 (34·4–42·3)	52·8 (47·2–58·5)
PEP courses completed	19·8 (17·3–22·2)	35·1 (31·4–38·9)	28·2 (25·3–31·2)	43·4 (38·7–48·1)
Total cost (US$)	1140 (1100–1260)	1110 (1070–1220)	935 (902–1030)	1300 (1250–1430)

Data are outcome in millions (95% PI), unless otherwise specified. Equivalent information on other scenarios (3 and 4a–c) is presented in the appendix. PI=prediction interval. PEP=post-exposure prophylaxis. DALYs=disability-adjusted life-years.

Projected vaccine requirements vary (figure 3 shows overall vaccine use, with four country-specific examples). Under improved post-exposure prophylaxis access, for example in Bangladesh, where intradermal regimens are used, vaccine demand incrementally increases, whereas in Kenya, where intramuscular regimens are used, vaccine requirements decrease because the same vaccine volume treats many more patients with intradermal vaccination. In Ethiopia, where nerve tissue vaccines are used, even larger reductions in vial use are expected.

Post-exposure prophylaxis prevents rabies at an average cost of $1021 per death averted and $52 per DALYs averted under the status quo (figure 2). With improved access, cost-effectiveness improves to $635 per death averted and $33 per DALYs averted in Gavi-67 countries and $605 per death averted and $32 per DALYs averted in Gavi-46 countries. Cost-effectiveness (appendix) varied geographically, and was lowest in Asia ($874 for deaths and $44 for DALYs) and highest in the Americas ($266 and $13). Incremental to the status quo, investment to improve post-exposure prophylaxis access would be highly cost-effective, averting 494 700 additional deaths, while reducing expenditure by $32·5 million (incremental cost-effectiveness ratio [ICER] –$66 per death averted or –$7 per DALYs averted).

Conversely, improving access to rabies immunoglobulin (scenario 3), in addition to vaccination is costly, requiring an additional $76·2 million (95% PI $73·0–85·0 million) between 2020 and 2035. Only marginal health gains are achieved, with about 100 additional deaths prevented and high uncertainty, resulting in a high ICER of almost $666 000 per death averted.

**Figure 4:**
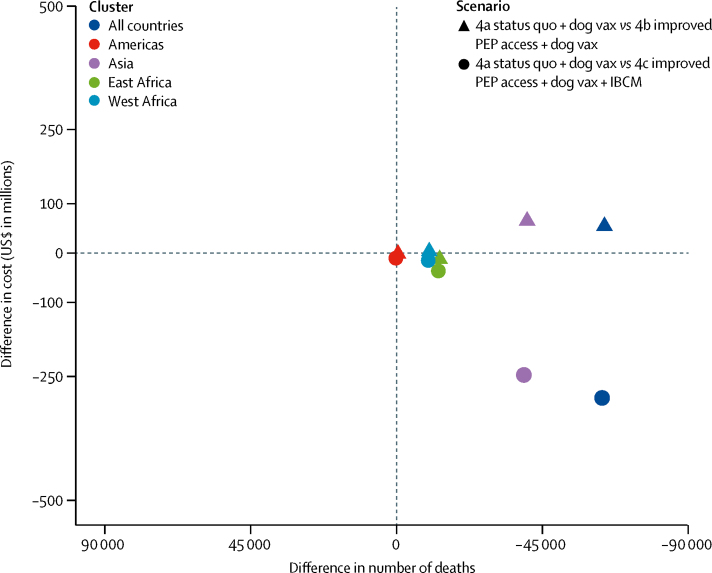
The cost-effectiveness plane showing the ICERs for dog vaccination scenarios Under dog vaccination (scenarios 4a–c) improved PEP access (scenarios 4b and 4c) all have greater health benefits and similar or lower costs. The differences between clusters relate to the size of the populations at risk. ICER=incremental cost-effectiveness ratio. IBCM=integrated bite case management. PEP=post-exposure prophylaxis. Vax=vaccination.

Under scenario 4, we assumed scaled-up dog vacci-nation programmes reduces the incidence of rabies exposures and deaths (Figure 2, Figure 3) to 328 000 deaths and 15 892 000 DALYs from 2020 to 2035. Improved access to post-exposure prophylaxis reduces the number of deaths to 266 000 and 12 847 000 DALYs, requiring 55·4 million vaccine vials from 2020 to 2035 (appendix) and remains cost-effective, albeit at a higher cost per death averted ($2307) and DALYs averted ($47; figure 2). Incremental to dog vaccination, investment in post-exposure prophylaxis results in lower mean costs and greater mean benefits (figure 4). In the third part of scenario 4 (4c), IBCM targets post-exposure prophylaxis to cases identified as bitten by suspect rabid dogs, rather than indiscriminately (panel), thereby controlling post-exposure prophylaxis demand as rabies incidence declines. IBCM should therefore be encouraged under all scenarios. Post-exposure prophylaxis requirements reduce to fewer than 21·3 million vials during 2020–35 and cost-effectiveness increases even as elimination is approached (figure 2; appendix).

Estimates of the rabies burden and cost-effectiveness of interventions are most affected by uncertainty in the incidence of rabid dog bites and the probability that exposure results in infection (appendix). Uncertainty in transmission affects rabies incidence and burden, whereas demographic uncertainty has a negligible effect. Improved dog vaccination coverage coordinated across regions more rapidly controls rabies, reducing the burden (appendix). Refined dog population estimates would change the magnitude of estimates of rabies burden and post-exposure prophylaxis use, but conclusions remain consistent across a plausible range of parameter values.

DALYs based on WHO frontier life expectancy in 2030 improved the overall cost per DALYs averted for scenario 2 from $33 to $30. Similar changes were observed for other scenarios.

Increasing vaccine vial costs from $5 to $10 increases the cost per death averted to $853, but post-exposure prophylaxis remains highly cost-effective. The ICER of rabies immunoglobulin exceeded $302 000 per death averted when vial costs were reduced from $45 to $20. Increasing the introductory grants to $500 000 did not affect our conclusions.

## Discussion

The burden of dog-mediated rabies is considerable; from 2020 to 2035 under the status quo, we estimate over 1 million deaths will occur in the 67 countries considered. Although post-exposure prophylaxis saves many lives, improved access, free of cost at point-of-care, and following the latest WHO recommendations for intradermal vaccination,^4^ would avert 1 388 000 deaths (ie, an additional 489 000 deaths) and 68 010 000 DALYs over this time period. This improvement in access is highly cost-effective at only $635 per death averted and $33 per DALYs averted and is feasible under current vaccine production with a switch to economical intradermal regimens through a Gavi investment. Interventions that avert one DALY for less than the national average annual per-capita income are considered highly cost-effective.^23^

Improving access to rabies immunoglobulin (mostly unavailable in rabies-endemic countries), in addition to vaccines, was not cost-effective. Benefits of rabies immunoglobulin were shown when used with nerve tissue vaccines,^24^ but this benefit is likely to be much less with the highly immunogenic purified cell culture and embryonated egg-based rabies vaccines recommended nowadays.^4^ Reported deaths among patients who initiate post-exposure prophylaxis appear to be related to treatment delays and poor compliance,^18^, ^25^ rather than a lack of rabies immunoglobulin availability.^26^ Investments to improve timely vaccine access are therefore likely to save many more lives than investment in rabies immunoglobulin.

WHO and partners launched the global strategic plan to end human deaths from dog-mediated rabies (Zero by 30) to eliminate human deaths from dog-mediated rabies through both mass dog vaccination and improved access to post-exposure prophylaxis.^5^ Cost-effectiveness of post-exposure prophylaxis decreases as elimination of canine rabies approaches, because genuine exposures become increasingly rare, whereas precautionary post-exposure prophylaxis provision continues. Nonetheless, our models suggest that in this context, improved access to post-exposure prophylaxis remains cost-effective (with a cost per DALYs averted less than gross domestic product per capita), particularly if implemented in conjunction with IBCM.^12^ Operational research on IBCM is warranted since it is a compelling strategy to curtail post-exposure prophylaxis demand while enhancing surveillance during the endgame.^27^

A major challenge for this study was the paucity of data available. Studies carried out in several rabies-endemic countries through the Gavi learning agenda allowed us to divide post-exposure prophylaxis demand by people bitten by healthy versus rabid animals, accounting for higher health seeking in wealthier settings, and variability driven by the size of dog populations. Because few dog population estimates were available, the accuracy of our projections are constrained, and results are notably sensitive to changes in health-seeking parameters. However, sensitivity analyses indicated that our conclusions regarding cost-effectiveness were robust even under uncertainties.

We made several simplifying assumptions. We assumed no country-specific variation in vaccine wastage from discarding opened vials, only rural–urban variation (appendix). Vaccine used per post-exposure prophylaxis course plateaus at throughputs exceeding 30 new patients per month;^21^ therefore, cost-effectiveness is unlikely to be greatly underestimated in the most populated settings, nor substantially overestimated in rural populations, because we do not account for patient clustering from bites by the same animal. Our assumptions regarding improved post-exposure prophylaxis access are supported by increased health seeking, access, and compliance observed with the introduction of free post-exposure prophylaxis in several countries (appendix).^18^ We also assumed incremental improvements in health seeking by victims of rabid bites over time, consistent with Gavi projections (and as used in models of other vaccine programmes)^28^ and data from countries that have invested in post-exposure prophylaxis access and free provision (Bhutan, Philippines, Sri Lanka). We did not model sustained increases in health seeking by people bitten by healthy dogs (potentially expected with heightened awareness associated with dog vaccination), thereby favouring the cost-effectiveness of improved post-exposure prophylaxis access. However, IBCM could reduce unnecessary post-exposure prophylaxis use and improve cost-effectiveness under all scenarios (we only modelled IBCM under dog vaccination, scenario 4c).^12^, ^13^, ^20^, ^29^ The effect and cost-effectiveness of post-exposure prophylaxis depends on current national strategies for provision (figure 3). We modelled these factors according to data gathered by WHO on post-exposure prophylaxis provision with timing aligned with the Global Strategic Plan. Although post-exposure prophylaxis access might be affected by spatial factors (eg, travel to clinics), we did not explicitly model this scenario, and instead assumed average improvements to access and provision (panel).

We applied our simulated trajectories of rabies incidence under dog vaccination irrespective of country, assuming 1% rabies incidence in unvaccinated dog populations. Dog vaccination has repeatedly been shown to rapidly reduce incidence,^30^, ^31^ although rabies often persists at low levels with incursions and localised wildlife reservoirs (not modelled here) hindering elimination.^32^ Moreover, socioeconomic factors influence progress by affecting the delivery of mass dog vaccination programmes.^33^ We assume coordinated implementation minimises incursions, thereby generating optimistic timelines for elimination. However, estimates of post-exposure prophylaxis requirements should not be greatly affected by this assumption given the considerable use of post-exposure prophylaxis for people bitten by non-rabid animals.^20^ While we considered the cost-effectiveness of post-exposure prophylaxis in the context of dog vaccination, we did not model dog vaccination costs; previous studies have shown dog vaccination to be very cost-effective.^29^, ^34^

In 2013, Gavi considered that rabies post-exposure vaccines could avert almost 200 000 future deaths between 2015 and 2030.^7^ We believe this outcome was an underestimate and our results suggest 489 000 additional deaths could be averted from an investment in improved post-exposure prophylaxis access. We have taken a more detailed approach using a decision tree informed by recent and relevant data, and we assume improvements in access, health seeking, and compliance.

Key barriers to rabies prevention are the limited supply and high costs of post-exposure prophylaxis, which are largely borne by patients. A Gavi investment in post-exposure prophylaxis should displace personal, but not government expenditure. Free post-exposure prophylaxis provision, with cofinancing between Gavi and national governments, would support the drive for universal health coverage to achieve the Sustainable Development Goals,^35^ and the consensus that rabies prevention should be a free public commodity.^5^ Gavi support would promote use of WHO prequalified vaccines and the 1-week intradermal regimen, standardise vaccine prices, and improve forecasting, procurement, and accountability. Stockouts, which are chronic in many rabies-endemic countries, could be prevented. Although post-exposure prophylaxis is outside the Expanded Programme on Immunization, efforts to strengthen this programme (particularly cold chain) greatly benefit post-exposure prophylaxis delivery. Thus, the potential of Gavi to shape the market, ensuring the availability or supply of WHO prequalified vaccines at affordable prices could be transformative for rabies prevention and would require a modest investment in the context of an annual budget in excess of $1 billion for vaccine programme disbursements.^36^

An investment in rabies vaccine in Gavi-eligible countries is likely to save many lives at a very low cost per death averted. Improving access to post-exposure prophylaxis alongside a switch to the newly-recommended intradermal 1-week regimen is feasible because vaccine vial requirements do not increase. Moreover, shifting costs from bite victims to donors and governments overcomes the primary barrier limiting access to life-saving vaccines and enables more efficient dose-sparing practices. To reduce an indefinite and escalating requirement for post-exposure prophylaxis, mass dog vaccination is essential to control and eliminate rabies in the reservoir population.
