# Supplementary material for: The potential effect of improved provision of rabies post-exposure prophylaxis in Gavi-eligible countries: a modelling study
Source: Lancet Infect Dis. 2019 Jan;19(1):102–11. doi: 10.1016/S1473-3099(18)30512-7 (PMC6300480; doi:10.1016/S1473-3099(18)30512-7)
Supplement: Supplementary appendix [file mmc1.pdf]

THE LANCET Infectious Diseases

Supplementary webappendix

This webappendix formed part of the original submission and has been peer reviewed. We post it as supplied by the authors.

Supplement to: WHO Rabies Modelling Consortium. The potential effect of improved provision of rabies post-exposure prophylaxis in Gavi-eligible countries: a modelling study. *Lancet Infect Dis* 2018; published online Nov 21. http://dx.doi.org/10.1016/S1473-3099(18)30512-7.

The potential effect of improved provision of rabies post-exposure prophylaxis in Gavi-eligible countries: a modelling study
WHO Rabies Modelling Consortium

Appendix

Supplementary Methods

1. Contributors to the WHO Rabies Modelling Consortium
2. Supplementary methods
    a. Biologically determined parameters
    b. Assumptions regarding improved access to PEP
    c. Epidemiological model
    d. Supplementary References
3. GATHER checklist
4. CHEERS checklist

Supplementary Figures

1. Full decision tree
2. Maps of countries in the study and data contributions
3. Comparison of estimated rabies deaths in 2020 by country
4. Comparison of health seeking, PEP provision and PEP compliance under different levels of PEP access.
5. Predictors of the incidence of bite patients presenting to health facilities from a range of countries
6. Univariate sensitivity analysis comparing DALYs, deaths and cost per DALY and death averted from 2020 to 2035 under the status quo (scenario 1), improved PEP access (scenario 2) and mass dog vaccination and improved PEP access (scenario 4b).
7. Model schematic describing rabies dynamics in dog populations and incidence of human rabies exposures.
8. Time series of dog rabies incidence under different modelling assumptions

Supplementary Tables

1. Rabies endemic countries included in analyses (Gavi-67), showing current Gavi eligibility in 2018 (Gavi-46), phase in Global Strategic Plan (GSP), and current PEP policy
2. Summary of the country specific parameters used and of the countries informing each parameter estimate
3. Country specific parameter values used in the decision tree
4. Cluster-average parameter values used in the decision tree
5. Biologically-defined constants used in the decision tree
6. Vial use assumptions for different PEP regimens
7. Burden of rabies by country and impacts of improved PEP access for each modelled scenario
8. Summary of model results across all Gavi-67 countries projected over 2020-2035 for the different scenarios.

Contributors to the WHO Rabies Modelling Consortium

The following institutions and individuals have contributed to the WHO Rabies Modelling Consortium.

UK
University of Glasgow, Institute of Biodiversity, Animal Health & Comparative Medicine, Glasgow, UK
Katie Hampson (PhD), Francesco Ventura (MSc), Rachel Steenson (MSc), Rebecca Mancy (PhD)
University of Cambridge, Department of Veterinary Medicine, Cambridge, UK
Caroline Trotter (PhD), Laura Cooper (MPhil)

World Health Organization
Department of the Control of Neglected Tropical Diseases, Geneva, Switzerland
Bernadette Abela-Ridder (PhD), Lea Knopf (DVM), Moniek Ringenier (MSc; intern)

Bhutan
National Centre for Animal Health, Department of Livestock, Ministry of Agriculture & Forests Serbithang, Babesa, Bhutan
Tenzin Tenzin (PhD)

Cambodia
Institut Pasteur International Network, Institut Pasteur in Cambodia, Epidemiology and Public Health Unit, Phnom Penh, Cambodia
Sowath Ly (PhD), Arnaud Tarantola (MD, MSc; *now Head, Epidemiology Unit, Institut Pasteur de Nouvelle-Calédonie, Paris*)

Chad
Centre de Support en Santé International, N'Djamena, Chad
RoneIngar Moyengar (MD)
Institut de Recherche en Elevage pour le Développement, N'Djamena, Chad
Assandi Oussiguéré (MD, MSc)

Côte d'Ivoire
Centre Suisse de Recherches Scientifiques, Abidjan, Côte d'Ivoire
Bassirou Bonfoh (PhD)

India
Department of Community Medicine, Kempegowda Institute of Medical Sciences (KIMS), Bangalore, India
D. H. Ashwath Narayana (MD)
Association for Prevention and Control of Rabies in India (APCRI), Bangalore, India
M. K. Sudarshan (MD)

Kenya
Zoonotic Disease Unit, Ministry of Health, Nairobi, Kenya
Athman Mwatondo (MSc)
Zoonotic Disease Unit, Ministry of Agriculture, Livestock and Fisheries, Nairobi, Kenya
Matthew Muturi (MSc)
Center for Global Health Research, Kenya Medical Research Institute, Kisumu, Kenya
Gati Wambura (MSc)

Madagascar
Ministère de la Santé Publique, Antananarivo, Madagascar
Glenn T Edosoa (MD, MPH)
Institut Pasteur de Madagascar, Antananarivo, Madagascar
Soa Fy Andriamandimby (MD, PhD), Laurence Baril (MD)

Mali
Laboratoire Central Vétérinaire, Bamako, Mali
Abdallah Traoré (PhD)

Philippines
Global Alliance for Rabies Control, Sta. Rosa, Philippines
Sarah Jayme (DVM, MVPHMgt)

RSA
MSD Animal Health, Johannesburg, RSA
Johann Kotzé (BVSc)

Sri Lanka
Rabies Treatment Unit, National Hospital, Colombo, Sri Lanka
Amila Gunesekera (MD)

Switzerland
Swiss Tropical & Public Health Institute, Basel, Switzerland
University of Basel, Basel, Switzerland
Nakul Chitnis (PhD), Jan Hattendorf (PhD), Mirjam Laager (PhD), Monique Lechenne (PhD), Jakob Zinsstag (PhD)

Tanzania
Ifakara Health Institute, Dar es Salaam, Tanzania
Joel Changalucha (MPH), Zac Mtema (PhD)
Sokoine University of Agriculture, Chuo Kikuu, Morogoro, Tanzania
Ahmed Lugelo (MSc)
Nelson Mandela African Institution of Science and Technology, Arusha, Tanzania
Kennedy Lushasi (MSc, *also Ifakara Health Institute, Dar es Salaam, Tanzania*)

Thailand
Department of disease control, Ministry of Public Health, Bangkok, Thailand
Onphirul Yurachai (DVM; *formerly O Sagarasaeranee*)

USA
Department of Ecology and Evolutionary Biology, Princeton University, Princeton, New Jersey, USA
Charlotte Jessica E. Metcalf (PhD), Malavika Rajeev (MSc)
Centers for Disease Control and Prevention (CDC), Atlanta, USA
Jesse Blanton (PhD), Galileu Barbosa Costa (PhD), Nandini Sreenivasan (MD), Ryan Wallace (DVM)
Kansas State University, Manhattan, Kansas, USA
Deborah Briggs (PhD)
Global Alliance for Rabies Control, Manhattan, Kansas, USA
Louise Taylor (PhD)
Paul G Allen School for Global Animal Health, Washington State University, Pullman, Washington, USA
Samuel M Thumbi (PhD, *Wellcome Trust Fellow at the Center for Global Health Research, Kenya Medical Research Institute, Kisumu, Kenya*)

Vietnam
National Institute of Hygiene and Epidemiology, Hanoi, Vietnam
Nguyen Thi Thanh Huong (MPH)

Appendix

A. Supplementary Methods

Data and code are on a Github Repository: github.com/katiehampson1978/rabies_PEP_access

Decision Tree Parameters

We used estimates of the risk of developing rabies following a bite by a probable rabid animal (P_{infect}) from a data set describing health utilisation and outcomes of bite victims derived from contact tracing in Tanzania.[1] Based on the health outcomes of bite victims who did not receive PEP, the probability of developing rabies following a bite was calculated. The overall probability of rabies transmission from a probable rabid animal bite was estimated based on the proportion of bite victims bitten on different body parts and the risk of developing rabies given the bite site. For the decision tree PSA we simulated this probability using a mixture model from these data.[1]

Individuals were assessed for the timeliness of PEP administration, where ‘timely’ PEP was defined as PEP received on the same day as the bite, and ‘late’ PEP was received 1 or more days after the bite. Individuals were also assessed for PEP completion, with 3 or more doses considered ‘complete’ and fewer doses considered ‘incomplete’. The probability of timely and complete PEP (>2 vaccine doses, without RIG) preventing rabies ($P_{prevent1}$) was estimated to be 1.00 (95% CI: 0.992-1.000), in line with zero deaths from 473 persons bitten by probable rabid dogs that completed timely PEP. The protection provided by imperfect (late or incomplete) PEP ($P_{prevent2}$) was based on a subset of these data. 14 deaths were identified from 1005 patients bitten by probable rabid animals that received imperfect PEP, i.e. $P_{prevent2}$ = 0.986 (95% CI 0.977-0.992). Nine of these deaths were attributable to delays in initiating PEP and five were associated with timely delivery of just one or two PEP doses only. Published data were extracted for nerve tissue vaccines (NTVs) with complete PEP defined as a minimum of 14 of the 17 daily vaccinations and incomplete PEP defined as <14 vaccinations.[2] The probability of preventing rabies given a complete NTV course ($P_{prevent3}$) was 0.99 (95% CI 0.97-0.99) and for an incomplete NTV course ($P_{prevent4}$) was 0.94 (95% CI 0.84-0.99). We only considered completeness of NTV courses because no information was provided on timeliness.

Evidence suggests that, even in the absence of RIG, the application of three timely high-quality cell culture or embryonated-egg vaccine doses is highly protective against rabies.[1,3] Only limited information is available on the protective effect of one or two timely vaccine doses (1 death from 51 bites by probable rabid dogs in Tanzania, versus 0 deaths from 124 patients with 3 timely doses). Other data indicates that a single timely vaccine dose is not completely protective (1 death following only a single timely 1st vaccination reported from Haiti, R *Wallace personal communication*). In general, delays in PEP administration are common and often conflated with incomplete PEP, as a result of high PEP costs and limited availability.[1,4,5] In the decision tree we assume that ‘imperfect’ PEP accounts for both the risk of death from incomplete PEP including patients in receipt of only one or two vaccine doses, and the risk of death from patients who received PEP (complete or incomplete) following a delay of at least one day. Although very rare, rabies deaths may occur despite timely and adequate PEP in the case of direct virus inoculation into a nerve or breakdown of the cold chain.

To estimate baseline health seeking for healthy dog bites ($p_{seek|healthy}$) we compiled data on reported bite incidence from health facilities from a range of countries. Using a GLMM we identified that bite patient incidence increased with the human development index (regression coefficient 5.4, Standard Error (SE) 2.16, p<0.0001), decreased with the urban proportion of the population (coefficient -3.1, SE 1.5, p<0.05) and increased with free access to PEP (coefficient 1.1, SE 0.44, p<0.05) (Figure S4). We adjusted our estimate of rabies exposure incidence by health seeking behaviour using country or cluster estimates of $p_{seek|rabid}$ to estimate the incidence of patients presenting to facilities due to rabies exposures. Using the data on bite patient incidence we estimated the proportion that were expected to result from rabid animals versus healthy animals to generate predictions of $p_{seek|healthy}$ from our regression together with estimated human and dog populations in each country.

We used country-specific data to inform the baseline probability (scenario 1 status quo) that rabid dog bite victims sought care and that on seeking care bite patients received and completed PEP (Tables S2 & S3). We assumed that on introduction of improved access, the probability of bite victims seeking, receiving and completing PEP all increased by probability 0.1 (Table 1). Following the introduction of improved PEP access, we also assumed incremental increases in these probabilities of 0.03 each year up to the maximum values reported in Table 1. We based our assumptions on data from studies where access to PEP was improved (Figure S4). In Tanzania PEP was provided for free and given intradermally at selected decentralized health facilities.[1] In Kenya and Chad PEP was provisioned for free at selected health facilities (GAVI Learning Agenda Studies). In Haiti, IBCM was undertaken with counselling to persuade those bitten by suspect rabid dogs to seek PEP.[6] In a mission hospital in Ethiopia PEP was provided for free as well as free accomodation for those travelling for more than one hour to access PEP.[7] Increases of this magnitude (0.1) were observed for health seeking, PEP provision and compliance in comparison to baseline or in contrast to areas without improved PEP access (Figure S4A). In some countries compliance was quite low even under improved PEP access, possibly due to awareness that bites were not due to rabid animals but instead due to healthy animals; an expected consequence of IBCM. We also generally observed higher health seeking and PEP provision in countries offering free or heavily subsidized PEP (Bhutan, Philippines, Madagascar, Cambodia),[8,9] in line with our assumptions (Figure S4B). Our analyses of determinants of incidence of bite patients presenting to health facilities further supports our contention that health seeking is higher when PEP is provided for free (Figure S5).

In our calculation of the cost of an investment to improve access to PEP, we assumed PEP costs are supported in all countries where governments do not currently provide free access to vaccine, including those where non-governmental organizations provide PEP or where governments subsidize vaccine but do not provide it free-of-charge (Table S1). Reports from several countries that provide free PEP, also suggest limited availability indicating additional support may be required to improve access, however this may be achieved through improved pricing and introductory grants that would come with Gavi investment.

We explored the influence of different parameters in the decision tree with a one-way sensitivity analysis. For the principal model outputs in scenario 1 (*status quo*), scenario 2 (*increased PEP access*) and scenario 4b (*mass dog vaccination with improved PEP access*) we ran a probabilistic sensitivity analysis (PSA) taking 1000 draws from the parameter distributions (based on 95% confidence intervals) to generate a 95% prediction interval (PrI). For each of these three scenarios, the univariate sensitivity analyses showed that the rabies burden and cost-effectiveness of interventions were most affected by uncertainty in the probability that PEP prevents disease and that exposure results in infection (Figure S6); reduced vaccine efficacy increases deaths while behavioural changes such as more prompt and thorough wound washing could reduce deaths. Generally, for the scenarios with improved PEP access the uncertainty in rabies burden reduced for all parameters. Under mass dog vaccination the sensitivity analyses indicated greater uncertainty in estimates of PEP cost-effectiveness associated with all parameters except for the incidence of bites by healthy dogs. The influence of the key intervention parameters (P_{seek}, $P_{receive}$, $P_{complete}$) was also explored for low and high alternatives (Table 2).

Dynamic Transmission model

To characterise rabies dynamics, we used a fully stochastic model parameterised from data on natural rabies infections in domestic dogs in Tanzania adapted from previously published models.[10] This comprised an individual-based model (IBM) of rabies transmission (Figure S7). Rabies transmission or susceptibility in dogs is neither age- nor sex-dependent; however, since domestic dog populations have high birth and death rates, and resulting population turnover rapidly reduces population immunity from vaccination, dog demography was included. We simulated demographic processes (births and deaths) using a Gillespie algorithm.

We fitted the IBM using Approximate Bayesian Computation to rabies incidence data for 2002-2015 from Serengeti district, Tanzania (dog population ~60,000) to characterise the functional form and spatial dependency of transmission. We implemented village-level dog vaccination campaigns within the model according to data on the location, timing and numbers of dogs vaccinated in Serengeti District. The modelling assumptions are illustrated in Figure S7. The model with the best fitting parameters corresponded to largely density-independent transmission with approximately 50 incursions per year (although the majority of these did not lead to secondary cases). The performance of post-hoc projections given initial conditions provided confidence that rabies dynamics were fully captured. Key life-history parameters for rabies (R_0, incubation period, infectious period, duration of vaccine-induced immunity, *per capita* exposures) are expected to be consistent across populations[11] assuming the use of high quality vaccines and analyses of epidemiological data including outbreak trajectories from a range of dog densities and landscapes are consistent with the simulated dynamics.[10-12]

Simulations from this model in the absence of mass dog vaccination showed that an average incidence of approximately 1% was necessary in order to maintain transmission. Typical confidence intervals were +/- 25% of the point estimate. Rather than use exact values from this

(currently unpublished) model that is specific to Tanzania, we used the model to characterise the incidence at 1% (range 0.75% to 1.25%) and used this in scenarios 1-3 of the decision tree.

For the purposes of sensitivity analysis and robustness testing, we considered the following alternative conditions: increasing and decreasing contact rates by 5% with respect to baseline and three realistic levels of vaccination coverage with baseline dog population turnover (Figure S8). In Gavi-eligible countries we expect dog lifespans to generally be short. For the baseline dog population turnover we therefore assumed a mean life expectancy of 25.8 months as estimated in Tanzania.[13] We also investigated a slower rate of dog turnover. We computed turnover for four communities in South Africa and Indonesia as weighted averages of months until loss from cohort, corrected for starting age.[14] In these four communities, dogs lived longer than in Tanzania. We used the community with the slowest turnover, Antiga (Bali, Indonesia) as an alternative, with mean life expectancy of 35.8 months.

For scenario 4, we applied annual mass dog vaccination campaigns commencing after month 36 of the simulation. The three alternative coverage levels that we investigated consisted of the observed vaccination data recorded for Serengeti district, a low coverage alternative using vaccination data from a year when dog vaccinations campaigns were not well implemented (2005), and a high coverage alternative from a year with well implemented campaigns (2015). For these alternatives, post-campaign coverage was 52% (baseline), 35% (low coverage) and 60% (high coverage) based on post-vaccination transect estimates. We assumed the use of high quality dog rabies vaccines providing lifelong immunity (>2.5 years) administered to dogs of all ages and health.[15] Declines in population immunity were therefore driven by population turnover rather than waning of vaccine-induced immunity. We incorporated spatial heterogeneity in coverage repeated over campaigns, as hard-to-reach populations tend to be consistently missed during campaigns. We assumed that annual campaigns preferentially target dogs that are already vaccinated, in line with anecdotal evidence, leading to conservative coverage levels. To simulate the effect of coordinated vaccination across contiguous populations we modelled a declining rate of incursions with time, using a three-month lag and a cubic function of the vaccinated-to-unvaccinated case ratio (i.e. ratio of number observed under vaccination relative to the unvaccinated baseline) in the preceding 45-day window. We simulated 100 realisations for each alternative condition and used the resulting trajectories in scenario 4 of the decision tree.

We examined the sensitivity of the epidemiological model to uncertainty in parameters. Uncertainty in transmission had a large effect on the incidence of dog rabies whereas the effect of demographic uncertainty, specifically the turnover of dog populations, was negligible (Figure S8). Improved dog vaccination coverage coordinated across regions to prevent incursions more rapidly controlled dog rabies (Figure S8) whereas lower coverage and lack of coordination prolonged rabies persistence. This would be expected given incursions from neighbouring countries or poor/patchy coverage in part of a country. These changes to the epidemiological model would translate in corresponding increases in human rabies deaths and use of PEP. Refined dog population estimates would also affect the magnitude of estimates of rabies burden and PEP use and the lack of data from different countries on the size of dog populations limits

our ability to more accurately project the impact of interventions (both dog vaccinations and PEP access). Nonetheless, our conclusions remain consistent across the plausible range of parameter values that we modelled.

Supplementary References

1. Changalucha, J. *et al.* The need to improve access to rabies post-exposure vaccines: lessons from Tanzania. *Vaccine* (in review).
2. Beyene, T. J., Mourits, M. C. M., Kidane, A. H. & Hogeveen, H. Estimating the burden of rabies in Ethiopia by tracing dog bite victims. *PLOS ONE* **13**, e0192313, doi:10.1371/journal.pone.0192313 (2018).
3. Tarantola, A. *et al.* Intradermal Rabies Post-exposure prophylaxis can be abridged with no measurable impact on clinical outcome in Cambodia, 2003-2014. (in review).
4. Sambo, M. *et al.* The Burden of Rabies in Tanzania and Its Impact on Local Communities. *PLoS Negl Trop Dis* **7**, e2510 (2013).
5. Tarantola, A. *et al.* Rabies Postexposure Prophylaxis Noncompletion After Dog Bites: Estimating the Unseen to Meet the Needs of the Underserved. *American Journal of Epidemiology* **187**, 306-315, doi:10.1093/aje/kwx234 (2018).
6. Etheart, M. D. *et al.* Effect of counselling on health-care-seeking behaviours and rabies vaccination adherence after dog bites in Haiti, 2014-15: a retrospective follow-up survey. *Lancet Global Health* **5**, E1017-E1025, doi:10.1016/s2214-109x(17)30321-2 (2017).
7. Ramos, J. M. *et al.* Epidemiology of animal bites and other potential rabies exposures and anti-rabies vaccine utilization in a rural area in Southern Ethiopia. *Ann Agric Environ Med* **22**, 76–79, doi:10.5604/12321966.1141372 (2015).
8. Rajeev, M. *et al.* Rabies exposures and post-exposure prophylaxis provisioning in the Moramanga District, Madagascar. *Vaccine* (in review).
9. Ly, S. *et al.* Rabies Situation in Cambodia. *PLoS Negl Trop Dis* **3**, e511. doi:510.1371/journal.pntd.0000511 (2009).
10. Townsend, S. E. *et al.* Designing Programs for Eliminating Canine Rabies from Islands: Bali, Indonesia as a Case Study. *Plos Neglected Tropical Diseases* **7**, e2372, doi:10.1371/journal.pntd.0002372 (2013).
11. Hampson, K. *et al.* Transmission Dynamics and Prospects for the Elimination of Canine Rabies. *Plos Biology* **7**, e1000053, doi:10.1371/journal.pbio.1000053 (2009).
12. Bourhy, H. *et al.* Revealing the Micro-scale Signature of Endemic Zoonotic Disease Transmission in an African Urban Setting. *Plos Pathogens* **12**, doi:10.1371/journal.ppat.1005525 (2016).
13. Czupryna, A. M. *et al.* Ecology and Demography of Free-Roaming Domestic Dogs in Rural Villages near Serengeti National Park in Tanzania. *PLOS ONE* **11**, e0167092, doi:10.1371/journal.pone.0167092 (2016).
14. Morters, M. K. *et al.* The demography of free-roaming dog populations and applications to disease and population control. *Journal of Applied Ecology* **51**, 1096-1106, doi:10.1111/1365-2664.12279 (2014).

15. Davlin, S. L. & VonVille, H. M. Canine rabies vaccination and domestic dog population characteristics in the developing world: A systematic review. *Vaccine* **30**, 3492-3502, doi:10.1016/j.vaccine.2012.03.069.
16. Hampson, K. *et al.* Estimating the Global Burden of Endemic Canine Rabies. *Plos Neglected Tropical Diseases* **9**, e0003786, doi:10.1371/journal.pntd.0003709 (2015).
17. Del Rio Vilas, V. J. *et al.* Tribulations of the Last Mile: Sides from a Regional Program. *Frontiers in Veterinary Science* **4**, doi:10.3389/fvets.2017.00004 (2017).
18. WHO, FAO, OIE & GARC. Zero by 30: The global strategic plan to end human deaths from dog-mediated rabies by 2030. (http://www.who.int/rabies/United_against_Rabies/en/ 2018).
19. APCRI. Gavi Learning Agenda Project report APCRI-INDIA. (2018).
20. Hampson, K. *et al.* Modelling to inform prophylaxis regimens to prevent human rabies. *Vaccine* (in review).

B. Supplementary figures

Figure S1: Full diagram of decision tree. *Gold shaded nodes incur costs. This tree assumes the use of high-quality cell culture vaccines. In Ethiopia where Nerve Tissues Vaccines (NTVs) are used, the probability that a complete or an incomplete NTV course prevents rabies is determined by $P_{prevent3}$ and $P_{prevent4}$, respectively.*

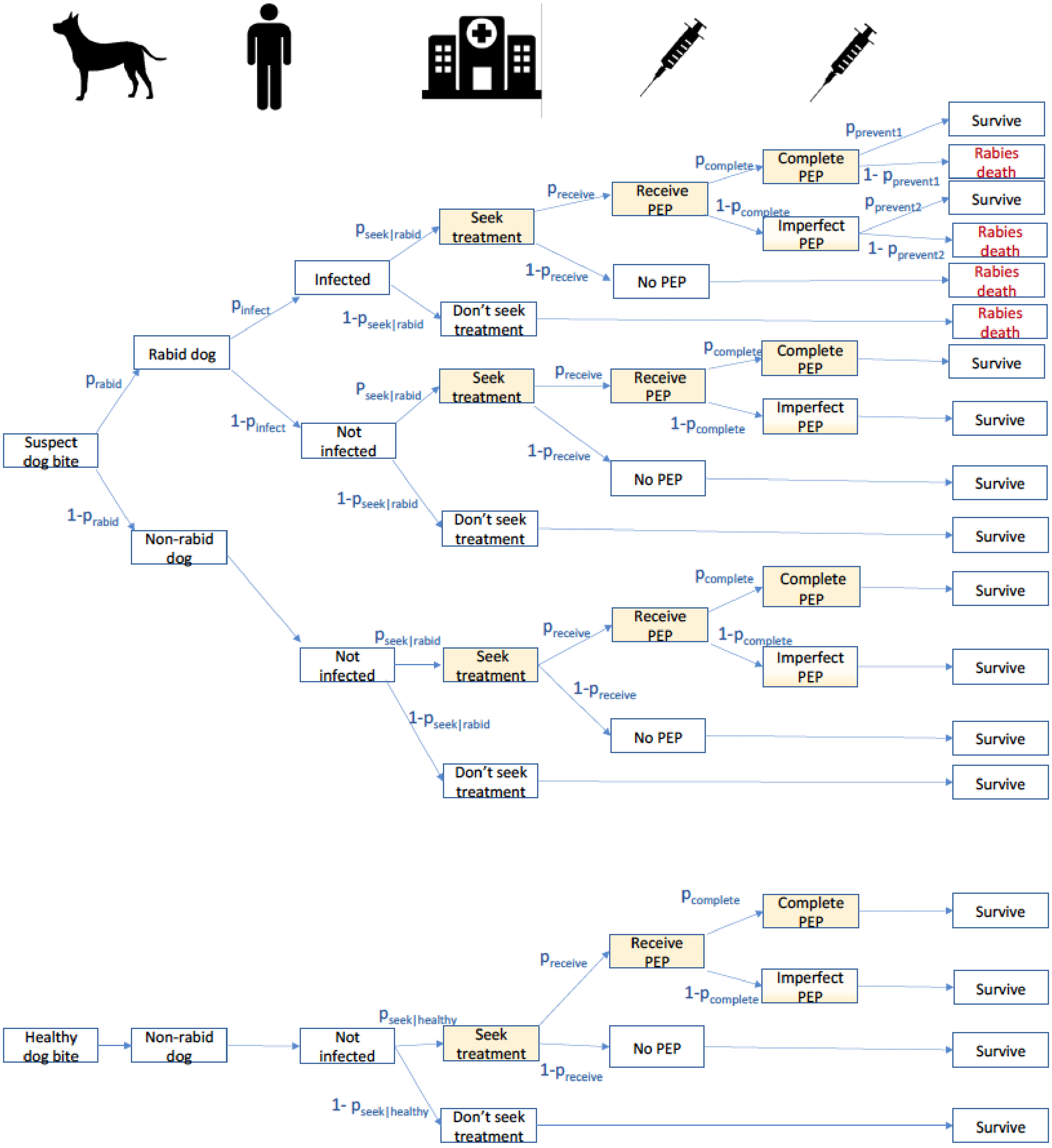

Figure S2: Map showing (A) countries modelled in the study and (B) countries that contributed data. *Countries that were modelled are shaded by cluster in A, with countries that were previously Gavi-eligible (Gavi-67) lightly shaded, and those Gavi-eligible in 2018 (Gavi-46) shaded darker. Countries that contributed data are shaded in B, including some that are not Gavi-eligible (Thailand, Philippines) and were not modelled in the study.*

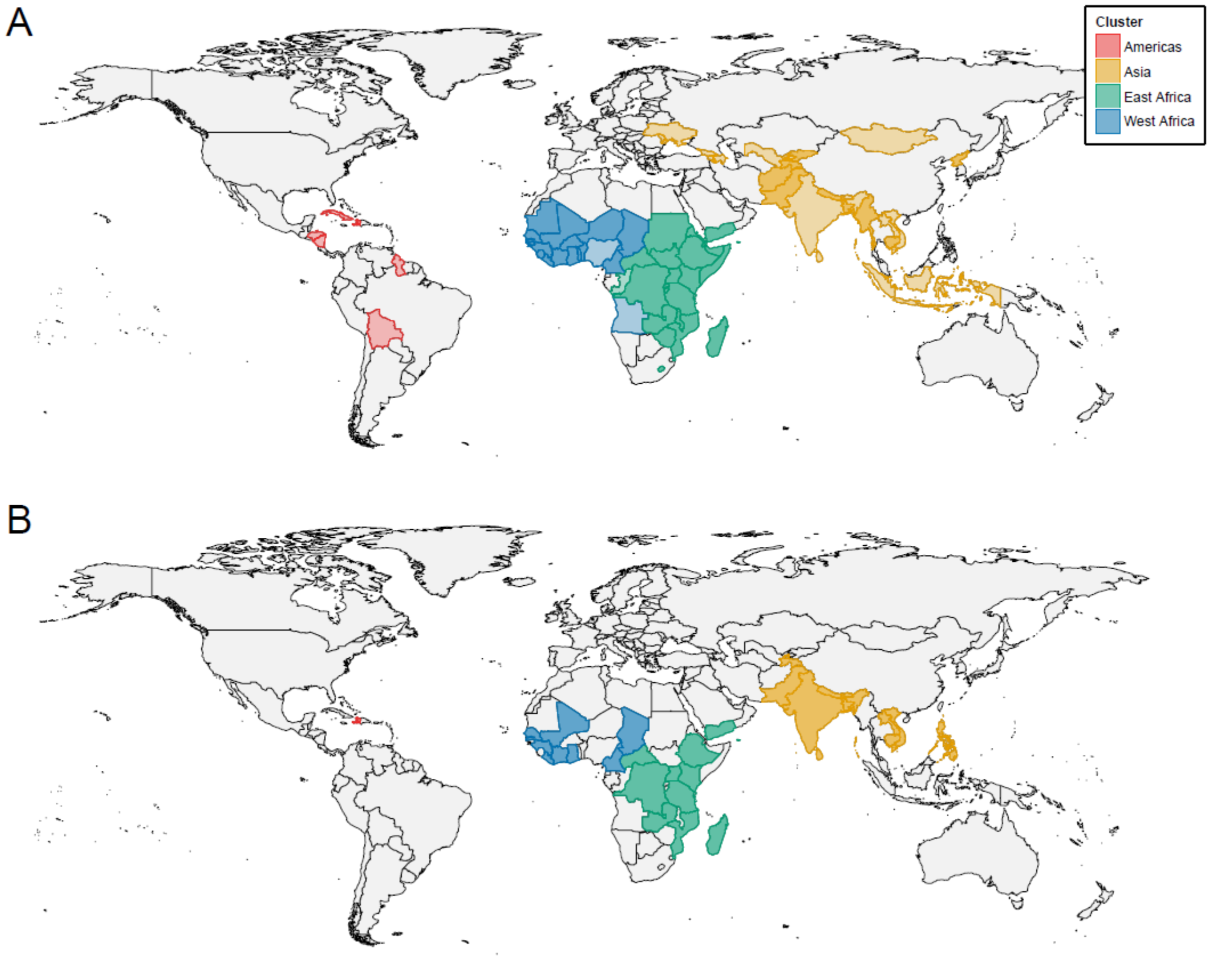

Figure S3: Comparison of estimated rabies deaths and 95% prediction intervals in 2020 by country. *Previous burden estimates,[16] shown in red, were based on data from 2010 and adjusted for population growth countries and compared to estimates from the current study (blue) for all Gavi-67 countries. This study may overestimate deaths in countries in Latin America where dog vaccination programmes are established.[17]*

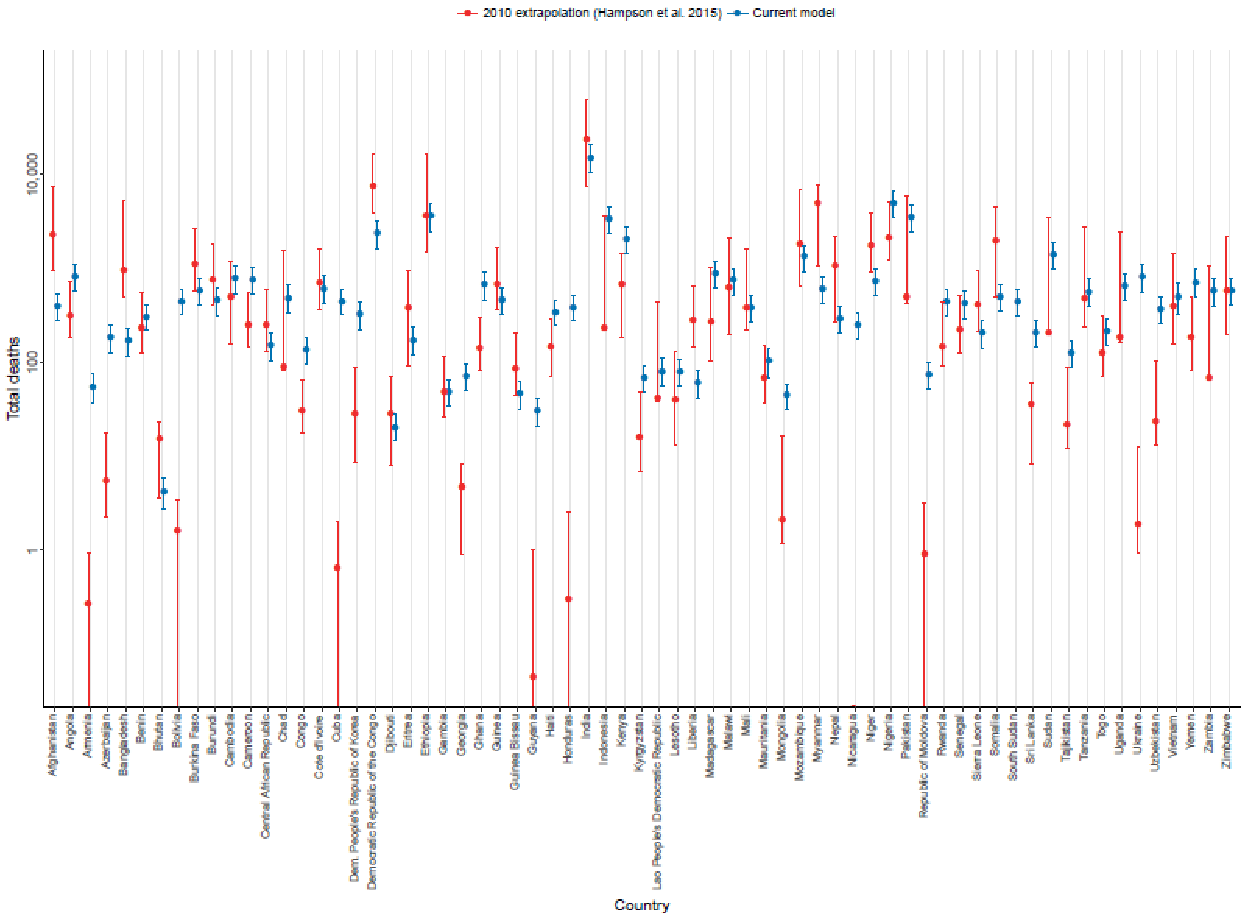

Figure S4: Comparison of health seeking, PEP provision and PEP compliance under different levels of PEP access. *Comparing estimates in A) settings following interventions to improve access to PEP and B) in countries where PEP is provided for free or is heavily subsidised versus countries whether patients must pay for PEP. Further details of studies are provided in the Appendix (Supplementary Methods - Decision tree parameters).*

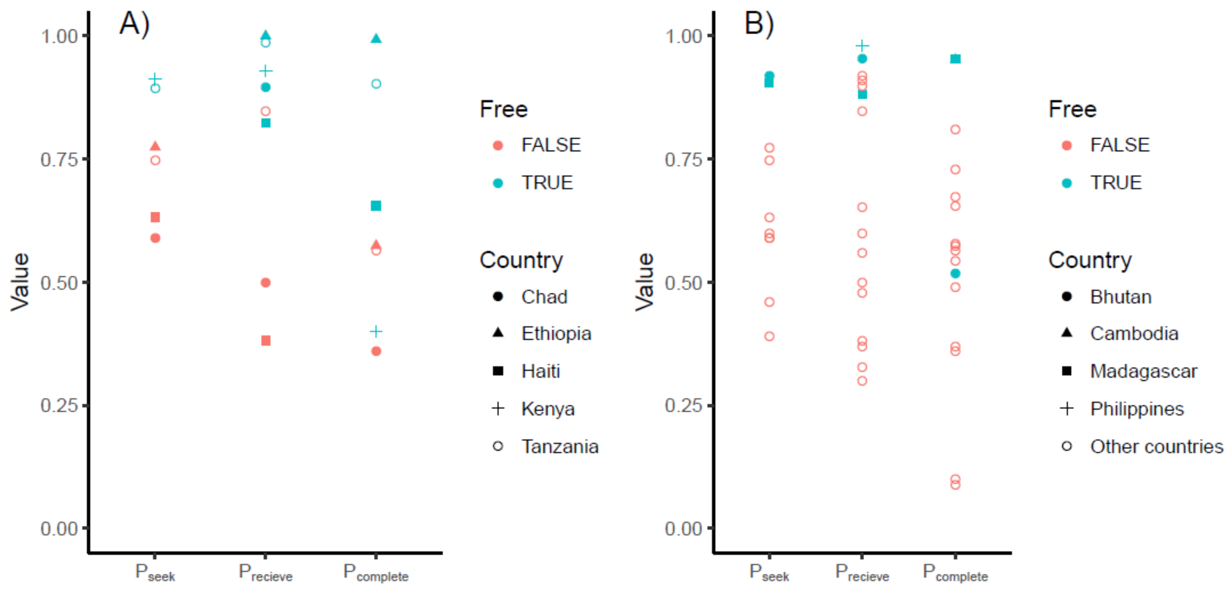

Figure S5. Predictors of the incidence of bite patients presenting to health facilities from a range of countries. *Bite patient incidence predicted from our regression coefficients is shown in relation to access to PEP (provided free of charge versus paid for by the patient) and national metrics for A) the Human Development Index and B) the proportion of the population in urban settings. Envelopes show 95% Confidence intervals.*

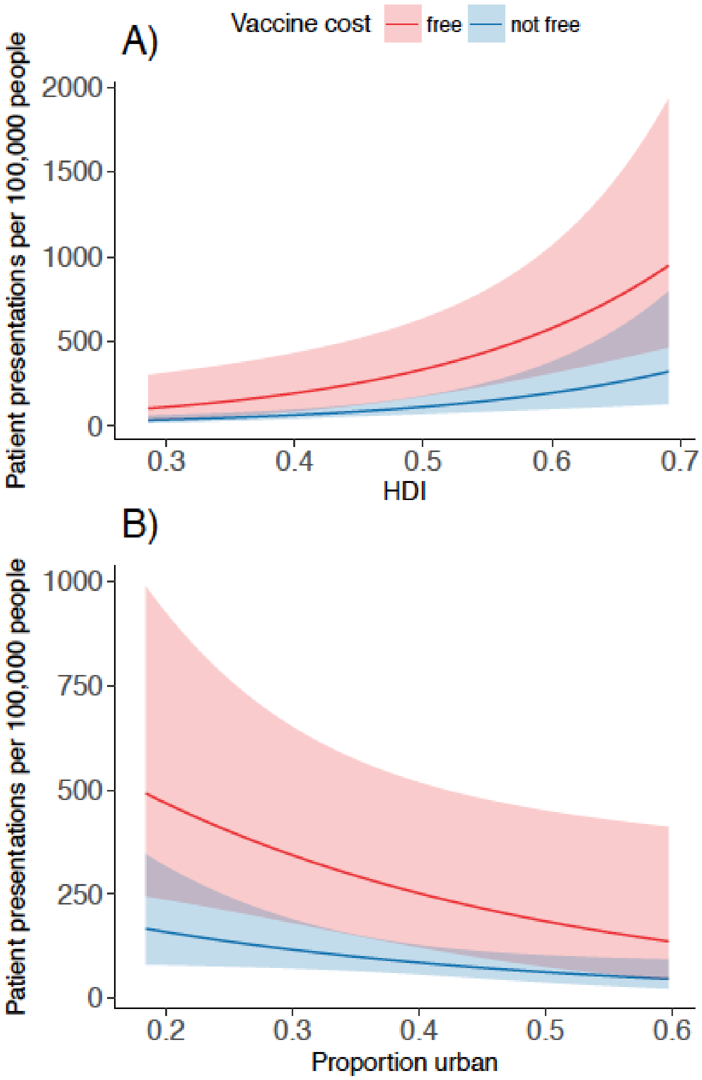

Figure S6: Univariate sensitivity analysis comparing mean outcomes and 95% Prediction Intervals from 2020 to 2035 under the status quo (scenario 1), improved PEP access (scenario 2) and mass dog vaccination and improved PEP access (scenario 4b). *A) DALYs (undiscounted); B) deaths (undiscounted); C) cost per DALY averted (discounted) and D) cost per death averted (discounted) from 2020 to 2035. Estimates are for Gavi-67 countries. For the univariate sensitivity analysis all four $P_{prevent}$ parameters (for complete and timely PEP, incomplete and timely PEP, and complete NTV use and incomplete NTV use) were explored together.*

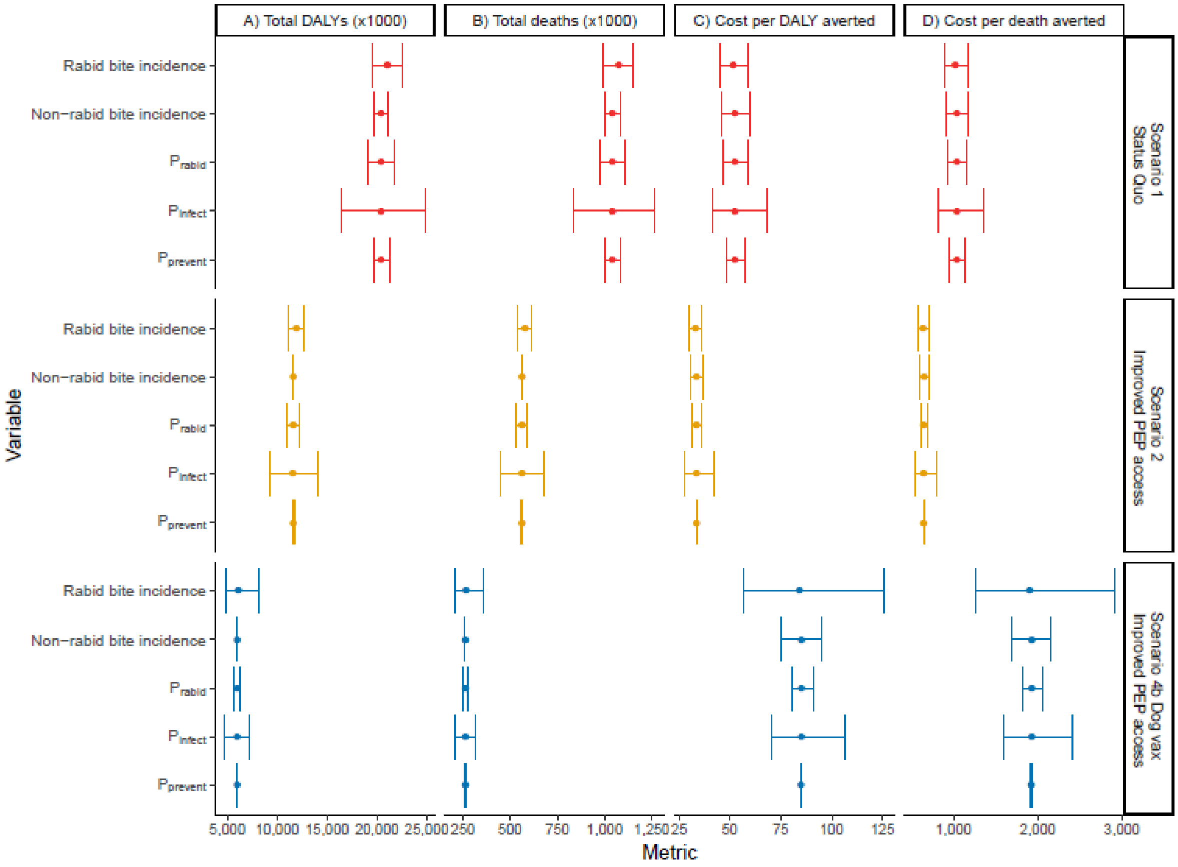

Figure S7: Model schematic describing rabies dynamics in dog populations and incidence of human rabies exposures.

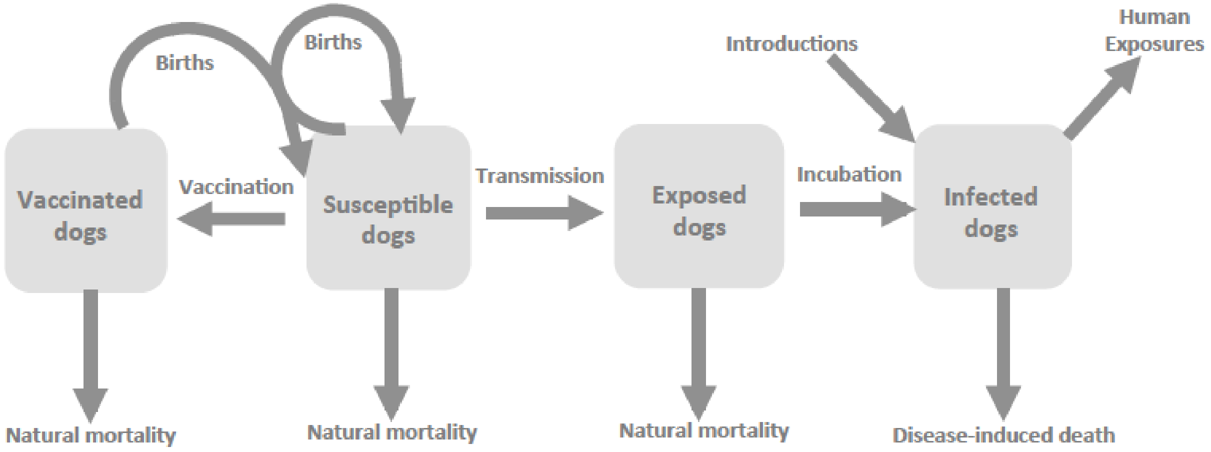

Figure S8: Time series of dog rabies incidence under different modelling assumptions. *A) Baseline with no dog vaccination (scenarios 1-3, light grey), versus coordinated mass dog vaccination from year 2 (scenario 4), versus dog vaccination with ongoing incursions from neighbouring areas (for sensitivity analyses); B) Baseline with no dog vaccination compared to high and low transmission; C) Coordinated mass dog vaccination compared to high and low vaccination coverage alternatives and D) Coordinated dog vaccination with fast versus slow turnover of the domestic dog population. Median incidence and 95% prediction intervals are shown (grey envelopes).*

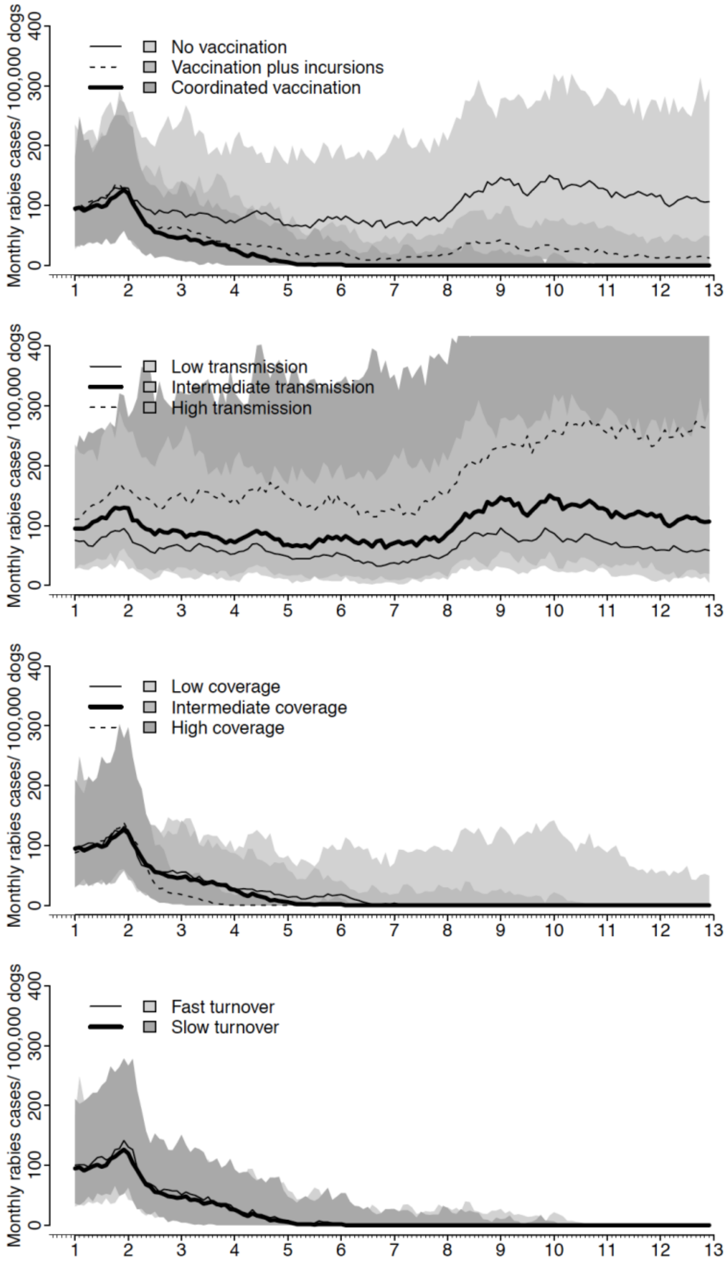

C. Supplementary tables

Table S1: Rabies endemic countries included in analyses (Gavi-67), showing current Gavi eligibility in 2018 (Gavi-46), phase in Global Strategic Plan (GSP),[18] and current PEP policy*. Although Gavi-eligible, the following countries were excluded from analyses as rabies is not endemic: Comoros, Kiribati, Papua New Guinea, Sao Tome and Principe, Solomon Islands and Timor-Leste. Nicaragua and Guyana are not included in the GSP while Honduras and Cuba are GSP Phase II countries,[18] however they were all modelled as Phase I countries since their established dog vaccination programmes have already reduced rabies incidence. We indicate government policy for PEP provision as reported to WHO. *In Madagascar, PEP is provided to patients for free courtesy of Institut Pasteur Madagascar, while in Cambodia, PEP is provided to patients at a subsidized rate by Institut Pasteur. In countries where the Ministry of Health policy is to provide PEP for free, availability is typically limited, with a few exceptions, such as Sri Lanka and Bhutan. To estimate the cost of a Gavi investment, we assumed that it would cover the cost of PEP in all countries where governments do not currently provide PEP for free (i.e. the patient pays the full cost or a subsidized cost).*

**Country**	**Cluster**	**Gavi-46**	**Gavi phase**	**GSP phase**	**PEP policy**
Afghanistan	asia	Yes	Initial self-financing	II	Free
Angola	west africa	No	Fully self-financing	II	Free
Armenia	asia	No	Fully self-financing	III	
Azerbaijan	asia	No	Fully self-financing	II	
Bangladesh	asia	Yes	Preparatory transition phase	I	Free
Benin	west africa	Yes	Initial self-financing	I	Patient pays
Bhutan	asia	No	Fully self-financing	I	Free
Bolivia	americas	No	Fully self-financing	I	
Burkina Faso	west africa	Yes	Initial self-financing	II	
Burundi	east africa	Yes	Initial self-financing	II	
Cambodia	asia	Yes	Preparatory transition phase	I	Subsidized*
Cameroon	west africa	Yes	Preparatory transition phase	II	Patient pays
Central African Republic	east africa	Yes	Initial self-financing	III	Free
Chad	west africa	Yes	Initial self-financing	II	Subsidized
Congo	east africa	No	Fully self-financing	II	
Cote d'Ivoire	west africa	Yes	Preparatory transition phase	II	Patient pays
Cuba	americas	No	Fully self-financing	II	Free
Dem. People's Republic of Korea	asia	Yes	Initial self-financing	III	
Democratic Republic of the Congo	east africa	Yes	Initial self-financing	III	Patient pays
Djibouti	east africa	Yes	Preparatory transition phase	II	Patient pays
Eritrea	east africa	Yes	Initial self-financing	II	
Ethiopia	east africa	Yes	Initial self-financing	I	Subsidized
Gambia	west africa	Yes	Initial self-financing	II	

Georgia	asia	No	Fully self-financing	I	Free
Ghana	west africa	Yes	Preparatory transition phase	I	Free
Guinea	west africa	Yes	Initial self-financing	II	Patient pays
Guinea Bissau	west africa	Yes	Initial self-financing	II	Free
Guyana	americas	No	Fully self-financing		
Haiti	americas	Yes	Initial self-financing	I	Patient pays
Honduras	americas	No	Fully self-financing	II	
India	asia	No	Accelerated transition phase	II	subsidized (75%)[19]
Indonesia	asia	No	Fully self-financing	II	
Kenya	east africa	Yes	Preparatory transition phase	I	Patient pays
Kyrgyzstan	asia	Yes	Preparatory transition phase	II	
Lao People's Democratic Republic	asia	No	Accelerated transition phase	II	Patient pays
Lesotho	east africa	Yes	Preparatory transition phase	I	Free
Liberia	west africa	Yes	Initial self-financing	I	Free
Madagascar	east africa	Yes	Initial self-financing	II	Subsidized*
Malawi	east africa	Yes	Initial self-financing	I	
Mali	west africa	Yes	Initial self-financing	I	Patient pays
Mauritania	west africa	Yes	Preparatory transition phase	III	
Mongolia	asia	No	Fully self-financing	II	Free
Mozambique	east africa	Yes	Initial self-financing	II	Free
Myanmar	asia	Yes	Preparatory transition phase	II	
Nepal	asia	Yes	Initial self-financing	II	Free
Nicaragua	americas	No	Accelerated transition phase		
Niger	west africa	Yes	Initial self-financing	III	Patient pays
Nigeria	west africa	No	Accelerated transition phase	II	Free
Pakistan	asia	Yes	Preparatory transition phase	II	Free
Republic of Moldova	asia	No	Fully self-financing	III	
Rwanda	east africa	Yes	Initial self-financing	II	
Senegal	west africa	Yes	Initial self-financing	II	
Sierra Leone	west africa	Yes	Initial self-financing	I	Patient pays
Somalia	east africa	Yes	Initial self-financing	III	Patient pays
South Sudan	east africa	Yes	Initial self-financing	III	
Sri Lanka	asia	No	Fully self-financing	I	Free
Sudan	east africa	Yes	Preparatory transition phase	III	Patient pays
Tajikistan	asia	Yes	Preparatory transition phase	III	
Tanzania	east africa	Yes	Initial self-financing	II	Patient pays
Togo	west africa	Yes	Initial self-financing	II	
Uganda	east africa	Yes	Initial self-financing	I	
Ukraine	asia	No	Fully self-financing	II	Free
Uzbekistan	asia	No	Accelerated transition phase	III	
Vietnam	asia	No	Accelerated transition phase	I	Patient pays

Yemen	east africa	Yes	Preparatory transition phase	III	
Zambia	east africa	Yes	Preparatory transition phase	II	
Zimbabwe	east africa	Yes	Initial self-financing	I	Free

Table S2: Summary of the country specific parameters used and of the countries informing each parameter estimate.

Parameter	Parameter Meaning	Number of countries contributing	Names of countries contributing
Bite incidence	Incidence of dog bites per population per year in health facilities	27	Bangladesh; Bhutan; Cambodia; Cameroon; Central African Republic; Chad; Cote d'Ivoire; Democratic Republic of the Congo; Ethiopia; Guinea; Haiti; India; Kenya; Lao People's Democratic Republic; Liberia; Mali; Mozambique; Nepal; Pakistan; Philippines; Sri Lanka; Tajikistan; Tanzania; Thailand; Uganda; Vietnam; Zambia
P_{rabid}	Probability that bite is from a rabid animal	12	Bhutan; Cambodia; Cameroon; Central African Republic; Chad; Democratic Republic of the Congo; Ethiopia; Kenya; Liberia; Tanzania; Vietnam; Yemen
$P_{seek\|rabid}$	Probability that bite victim will seek health care treatment (PEP)	9	Bhutan; Chad; Cote d'Ivoire; Ethiopia; Haiti; Mali; Tanzania; Uganda; Vietnam
$P_{receive}$	Probability that bite victim seeking treatment will actually receive PEP	15	Bhutan; Cameroon; Central African Republic; Chad; Cote d'Ivoire; Guinea; Haiti; Liberia; Mali; Mozambique; Pakistan; Philippines; Tajikistan; Tanzania; Uganda
$P_{complete}$	Probability that bite victim will complete full PEP course	15	Bhutan; Cambodia; Cameroon; Chad; Cote d'Ivoire; Ethiopia; Guinea; Haiti; India; Liberia; Mali; Pakistan; Tanzania; Thailand; Uganda
Dog population	Total dog population	11	Bangladesh; Bhutan; Cambodia; Cameroon; Chad; Haiti; India; Philippines; Tanzania; Uganda; Vietnam
Vaccine price per vial	Cost of vaccine vial	27	Bangladesh; Bhutan; Bolivia; Cambodia; Cameroon; Chad; Cote d'Ivoire; Democratic Republic of the Congo; Ethiopia; Ghana; Haiti; Honduras; India; Indonesia; Kenya; Lao People's Democratic Republic; Mali; Myanmar; Nepal; Nicaragua; Pakistan; Philippines; Sri Lanka; Tanzania; Thailand; Ukraine; Vietnam

Table S3: Country specific parameter values used in the decision tree:
github.com/katiehampson1978/rabies_PEP_access/blob/master/tables/country_data_Gavi.xlsx
For countries with no data, cluster values were used instead (Table S3), estimated as the average of the country values within each cluster.

Table S4: Cluster-average values used in the decision tree. *Sample size denotes the numbers of individuals contributing data to a parameter. Countries in each cluster are detailed in Table S1. Costs in East Africa exclude Ethiopia, where Nerve Tissue Vaccines (NTVs) are used.*

**Cluster**	**$P_{seek\|rabid}$ estimate**	**$P_{seek\|rabid}$ sample size**	**$P_{receive}$ estimate**	**$P_{receive}$ sample size**	**$P_{complete}$ estimate**	**$P_{complete}$ sample size**	**Vaccine price per vial (USD)**	**RIG price per vial (USD)**
Americas	0.632	68	0.382	68	0.655	68	41	145
Asia	0.76	1,200	0.754	33,497	0.753	41,167	13	41
East Africa	0.66	2,240	0.543	2,415	0.413	1,463	16	168
West Africa	0.523	1,095	0.687	9,838	0.422	4,261	15	83

Table S5. *Biologically-defined constants.* *NTV - Nerve Tissue Vaccines. *assumes use of high quality cell-culture vaccine; **incomplete vaccination is defined as fewer than 3 doses given or late vaccination initiated ~1 days after exposure. ***Incomplete vaccination for NTVs assumes <14 injections, we did not consider timeliness of NTV administration. Further details of these parameter estimates are provided in the appendix.*

**Parameter**	**Probability**	**Value (95% CIs)**	**n**	**Rationale**
p_{infect}	Developing infection in the absence of PEP	0.165 (0.133-0.201)	2,877	Observational studies [13]
$p_{prevent1}$	Complete and timely vaccination prevents rabies*	1.000 (0.992-1.000)	473	Observational studies [13]
$p_{prevent2}$	Incomplete/ late vaccination prevents rabies**	0.986 (0.977-0.992)	1,005	Observational studies [13]
$p_{prevent3}$	Complete NTV prevents rabies	0.993 (0.973-0.998)	267	Observational studies [14]
$p_{prevent4}$	Incomplete NTV prevents rabies***	0.941 (0.841-0.98)	51	Observational studies [14]

Table S6: Vial use assumptions for different regimens. *For intradermal regimens we estimated vial use on the assumption of average throughput of 100 new bite patients per month in urban settings and 5 per month in rural settings.[20] We used estimates of vial use for patients who complete PEP and those who initiate PEP but discontinue vaccination without completing the course. For NTVs which are still used in Ethiopia, we based vial use on published data,[2] with assumptions detailed in the Supplementary methods.*

Setting	Regimen	Compliance	Vaccine vials/ patient	RIG vials/ patient
Rural	IM	Complete	4	NA
Urban	IM	Complete	4	0.32
Rural	Updated TRC	Complete	2.76	NA
Urban	Updated TRC	Complete	0.93	0.32
Rural	IPC	Complete	2.2	NA
Urban	IPC	Complete	0.67	0.32
Rural	NTV	Complete	14	NA
Urban	NTV	Complete	14	0.32
Rural	IM	Incomplete	2	NA
Urban	IM	Incomplete	2	0.32
Rural	Updated TRC	Incomplete	1.84	NA
Urban	Updated TRC	Incomplete	0.62	0.32
Rural	IPC	Incomplete	1.47	NA
Urban	IPC	Incomplete	0.45	0.32
Rural	NTV	Incomplete	9.33	NA
Urban	NTV	Incomplete	9.33	0.32

Table S7: Burden of rabies by country and impact of improved PEP provision for each scenario. *Country-specific burden estimates are available here:*

github.com/katiehampson1978/rabies_PEP_access/blob/master/tables/metric_summary_by_country.xlsx

Mean values are given with 95% confidence intervals in brackets for model outputs under Status Quo (scenario 1) and Improved PEP access (Scenario 2 base case).

Table S8. ***Summary of model results across all Gavi-67 countries projected over 2020-2035 for the different scenarios.*** *The mean and 95% prediction intervals of the burden of rabies (deaths and DALYs), the impact of PEP (deaths and DALYs averted), PEP courses initiated and completed, vials of vaccine used and costs (undiscounted), are shown under the status quo, improved access to rabies vaccine (base case - details in Table 1), provision of RIG, and under the dog vaccination scenarios (status quo, improved PEP provision and Integrated Bite Case Management).*

Outcomes (in millions)	Scenario 1 Status Quo (95% PrIs)	Scenario 2. base case - improved PEP access (95% PrIs)	Scenario 3. As in 2 + Provision of RIG (95% PrIs)	Scenario 4a Status Quo with dog vaccination (95% PrIs)	Scenario 4b. Dog vaccination + improved PEP access (95% PrIs)	Scenario 4c. As in 4b + IBCM (95% PrIs)
Rabies deaths	1.07 (0.852-1.32)	0.576 (0.453-0.711)	0.577 (0.455-0.709)	0.328 (0.224-0.471)	0.266 (0.190-0.366)	0.266 (0.188-0.364)
Rabies deaths averted	0.898 (0.704-1.11)	1.39 (1.09-1.72)	1.39 (1.09-1.71)	0.281 (0.193-0.404)	0.344 (0.226-0.510)	0.343 (0.222-0.509)
DALYs	52.1 (41.4-64.3)	27.9 (21.9-34.5)	28.0 (22.0-34.3)	15.9 (10.9-22.8)	12.8 (9.14-17.7)	12.8 (9.06-17.6)
DALYs averted	44.2 (34.7-54.6)	68.0 (53.30-84.3)	68.1 (53.6-83.6)	13.8 (9.48-19.8)	16.9 (11.1-25.1)	16.9 (10.9-25.0)
Vaccine vials used	73.5 (65.7-81.4)	73.8 (66.1-81.6)	73.8 (65.9-81.6)	49.1 (41.1-57.8)	55.4 (47.4-64.1)	21.3 (16.0-29.6)
RIG vials used	0.0 (0.0-0.0)	0.0 (0.0-0.0)	2.16 (1.93-2.38)	0.0 (0.0-0.0)	0.0 (0.0-0.0)	0.0 (0.0-0.0)
PEP courses initiated	27.8 (24.6-31.0)	45.2 (40.5-50.0)	45.2 (40.4-50.0)	20.4 (17.1-23.9)	33.0 (28.2-38.2)	11.8 (8.55-16.9)
PEP courses completed	19.8 (17.3-22.2)	35.1 (31.4-38.9)	35.2 (31.4-38.9)	14.9 (12.5-17.4)	25.6 (21.9-29.7)	8.87 (6.38-12.8)
Total Cost (USD)	1,140 (1,100-1,260)	1,110 (1,070-1,220)	1,200 (1,160-1,320)	717 (671-849)	794 (750-915)	342 (307-462)
